# Vitamin B_12_‐Functionalized Nanogels for pH‐, and Redox‐Responsive Doxorubicin Delivery

**DOI:** 10.1002/cbic.70580

**Published:** 2026-10-26

**Authors:** Serife Dagdelen, Paula D. Mestizo, Marcin Mackiewicz, Agnieszka Kurdyn, Ewa Augustin, Marcin Karbarz, Felix Zelder

**Affiliations:** ^1^ Faculty of Chemistry University of Warsaw Warsaw Poland; ^2^ Biological and Chemical Research Center University of Warsaw Warsaw Poland; ^3^ Department of Chemistry University of Zürich Zürich Switzerland; ^4^ Department of Molecular Biology, Institute of Biochemistry, Faculty of Biology University of Warsaw Warsaw Poland; ^5^ Department of Pharmaceutical Technology and Biochemistry, Faculty of Chemistry Gdańsk University of Technology Gdańsk Poland

**Keywords:** cancer cell, chemistry, cofactor, covalent bond, cytotoxicity, doxorubicin, drug delivery, in vitro, nanogel, vitamin B**_12_**

## Abstract

Rapidly proliferating cancer cells display an elevated demand for vitamin B_12_, rendering this bacterial‐derived cofactor an attractive targeting motif for nanotherapeutic drug delivery. Herein, we report a concise two‐step synthesis of a polymerizable 5′‐modified vitamin B_12_ ‐monomer that enables the covalent integration of B_12_ into pH‐ and redox‐responsive nanogels. The resulting nanogels efficiently encapsulate doxorubicin through electrostatic interactions between the cationic drug and the anionic nanogel network, while enabling stimuli‐responsive drug release under tumor‐relevant conditions. In vitro studies revealed that DOX@B_12_‐nanogels retain potent antiproliferative activity against HT‐29 colorectal cancer cells (IC_50_ = 0.878 ± 0.102 μM), while decreasing cytotoxicity toward healthy CCD 841 CoN cells by 34‐fold relative to non‐B_12_‐functionalized control nanogels. These findings establish covalently engineered vitamin B_12_‐nanogels as a promising platform for selective and stimuli‐responsive anticancer drug delivery.

## Graphical abstract

A polymerizable 5′‐modified vitamin B_12_‐monomer enables the covalent incorporation of B_12_ into pH‐ and redox‐responsive nanogels for targeted doxorubicin delivery. The nanogels exhibit glutathione‐triggered drug release, potent antiproliferative activity against HT‐29 cancer cells, and strongly reduced cytotoxicity toward healthy cells.

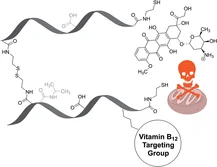

## 1 Introduction

The primary approaches for treating cancer involve surgery, chemotherapy, and radiation. Chemotherapy, particularly when combined with other therapies, remains widely used and effective [1]. However, traditional nontargeted chemotherapy has its limitations and often causes systemic side effects, and cancer recurrence rates remain high [2, 3]. To overcome these drawbacks, targeted therapy approaches strive to develop improved methods to deliver drugs directly to tumors using nano or microsized drug carriers. Pioneering drug carrier systems, including liposomes [4], polymeric micelles [5, 6], lipids [7], and hydrogels [8, 9], have already demonstrated improved precision and effectiveness in chemotherapy, with many already available on the market or in various stages of clinical trials. Notable discussions of these advancements are found in comprehensive reviews by Shi et al. [10] and Fan et al. [11], which detail the significant advances and ongoing challenges in the field of cancer nanomedicine, underscoring the critical role these systems play in advancing chemotherapy techniques.

Among such targeted drug‐delivery systems, hydrogels have garnered significant attention due to their unique physicochemical properties and versatility [12]. Composed of water and hydrophilic polymers, hydrogels and their nano and microparticle forms, known as nano and microgels, are particularly effective due to their high water content, porosity, and ability to protect encapsulated drugs against degradation in the bloodstream, efficient treatment with reduced systemic toxicity, improved drug solubility, and compatibility with a range of drugs [13, 14, 15, 16, 17, 18]. These properties make them excellent candidates for carrying and releasing cancer‐fighting agents such as doxorubicin, paclitaxel, and 5‐fluorouracil where they are needed in the body [19]. While nanogel technology has advanced, designing nanogels that achieve safe and controlled drug release remains a challenge. Much research has focused on creating specialized nanogels that respond to specific triggers within the tumor environment. These stimuli, whether endogenous, such as enzymes [20, 21], pH levels [22], reactive oxygen species (ROS) (i.e., dioxygen, O_2_) [23] or reducing environment (i.e., glutathione, GSH) [24, ‍^25^], adenosine‐5′‐triphosphate (ATP) [26] levels or exogenous sources like heat [27, 28], magnetism [29, 30], ultrasound [31], or light [32, ‍33], enable nanogels to target tumors more accurately, allowing for effective treatment with lower doses.

Improvements in the development of single‐stimuli‐responsive nanogels have stimulated research efforts to design multifunctional nanogels. In fact, only recently, multistimuli‐responsive nanogels have shown superior drug‐releasing properties at tumor sites, outperforming single‐response systems [34, 35, 36, 37, 38]. Unfortunately, the current generation of multistimuli‐responsive nanogels still often lacks tumor selectivity, relying on passive targeting mechanisms such as the enhanced permeability and retention (EPR) effect [39]. These approaches are limited by the heterogeneous vascularization of tumors and insufficient targeting precision, leading to suboptimal therapeutic outcomes [40, 41]. In a different approach, functionalized nanogels with antibodies or folate as targeting agents have also been developed to increase the capacity for targeted delivery of anticancer drugs to tumors [42, 43, 44, 45]. Although these approaches are promising, specific functional limitations of antibodies have been identified, including restricted penetration into solid tumor masses and limited activation or interaction with immune effector mechanisms [46]. In the case of folate‐functionalized nanogels, folic acid presents conjugation and formulation challenges due to its limited aqueous solubility and potential cytotoxicity at higher concentrations [47].

Vitamin B_12_ (**1**; Figure 1) is an attractive candidate due to its essential biological role and receptor‐mediated uptake via the transcobalamin II (TCII) transport system [48, 49]. Rapidly proliferating cells exhibit increased B_12_ demand [50, 51], and modification at the 5′‐ribose position preserves key structural features required for TCII recognition [52, 53, 54, 55]. These features make B_12_ a promising molecular targeting motif [56, 57].

**FIGURE 1:**
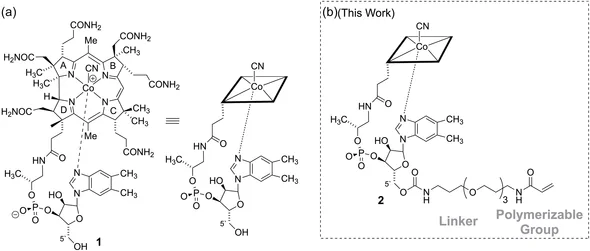
(a) Structural formula and schematic representation of vitamin B_12_ (**1**). The 5′‐position at the ribose subunit is indicated. (b) Schematic representation of the B_12_ building block **2**. The 5′‐position, polyethylene glycol linker and polymerizable acrylamide group are indicated.

Despite this potential, polymerizable B_12_ derivatives for covalent integration into polymer networks remain unexplored. Previous approaches have relied on noncovalent interactions or coordination to the cobalt center, which may suffer from limited stability and uncontrolled ligand substitution reactions [58, 59]. In other studies, chitosan‐based hydrogels were developed for cobalamin delivery [60].

Herein, we report a polymerizable B_12_‐monomer **2** (Figure ‍1b) that enables its covalent incorporation into nanogels (i.e., B_12_‐nanogel **3**) via radical copolymerization. This approach provides controlled integration of a structurally intact B_12_ unit into a pH‐ and redox‐responsive polymer network and enables encapsulation of doxorubicin (DOX@B_12_‐nanogel **4**) by electrostatic interactions.

## 2 Results and Discussion

A polymerizable B_12_‐monomer **2** was synthesized in two steps via modification at the 5′‐ribose position of B_12_ (**1**), introducing first a polyethylene glycol (PEG)‐based linker followed by the addition of an acrylamide functionality (Figure 1b, Schemes S1, and S2) [61]. This design enables covalent incorporation into polymer networks while maintaining the structural integrity of the vitamin in its “base‐on” form with an intramolecularly coordinated Co(III) dimethyl benzimidazole base. The LC–MS spectrum of the B_12_‐monomer (Figure S4) showed a single peak (*t*_ret_ = 2.0 min) for the [M  +  2H]^2+^ at *m*/*z* = 828.37, indicating the molecular formula C_77_H_112_CoN_16_O_19_P (*m*/*z*_calc_: 1655.74). Three bands at 362, 519, and 549 nm were observed by UV–vis analysis that are expected for the α‐, β‐, and γ‐bands of the corrin chromophore (Figure S3), and the ^1^H‐NMR spectrum (Figures S8–S12) showed a singlet at 5.89 ppm for H─C_10_ of the corrin ring and three indicative sets of doublets of doublets between 5.54 and 6.21 ppm for the terminal olefin moiety indicate a pure, covalent B_12_‐monomer **2** (Figure S12).

The B_12_‐monomer **2** was copolymerized with *N*‐isopropylacrylamide (NIPA), acrylic acid (AA), and *N*,*N*′‐bis(acryloyl)cystamine (BAC)—a disulfide‐containing crosslinker—to yield B_12_‐nanogel **3** as outlined in the Supporting Information. (Schemes 1 and S3). A control nanogel lacking B_12_ was prepared analogously (Scheme S4). UV–vis spectroscopy of B_12_‐nanogel (Figure 2a) suggests almost quantitative (92%) incorporation of **2** into polymer **3** by indicating the characteristic three absorption bands of the corrin ligand lacking in the spectrum of the control gel.

**SCHEME 1:**
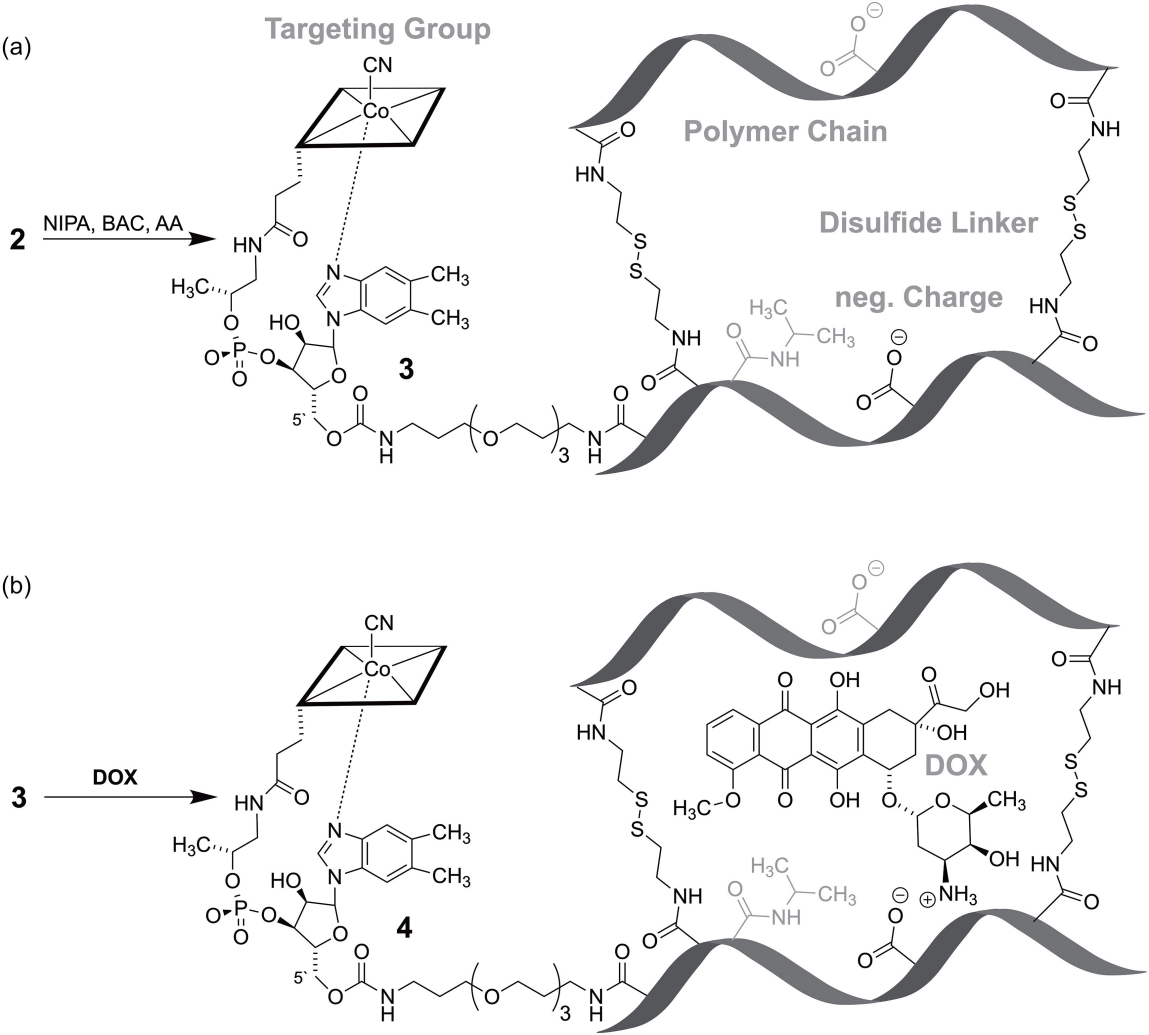
(a) Synthesis of the B_12_‐nanogel **3** from B_12_‐monomer **2** via radical copolymerization (NIPA: *N*‐isopropylacrylamide; BAC: *N*,*N*′‐bis(acryloyl)cystamine; AA: acrylic acid). (b) Loading of **3** with doxorubicin (DOX) to DOX@B_12_‐nanogel **4**.

**FIGURE 2:**
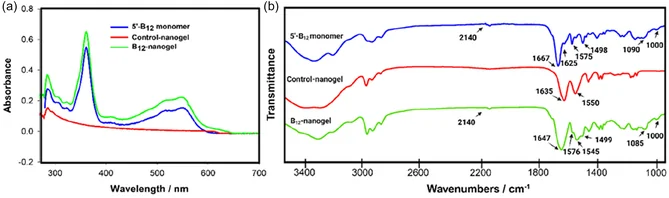
UV–vis absorption spectra (a) and FTIR spectra (b) of the B_12_‐monomer **2**, B_12_‐nanogel **3**, and control nanogel.

Further characterization of the B_12_‐nanogel **3** was performed by comparing its FTIR spectrum with the control nanogel and the B_12_‐building block **2** (Figure 2b). All three spectra show characteristic broad bands above 3000 cm^−1^, corresponding to O─H and N─H stretching vibrations. Bands in the range between 3000 and 2800 cm^−1^ are assigned to asymmetric and symmetric vibrations of the methyl and methylene groups [62]. An indicative signal at 2140 cm^−1^ is only present in the spectra of **2** and the B_12_‐nanogel but not in the spectrum of the control nanogel lacking the targeting group. It is characteristic for the C≡N stretch of the cobalt‐coordinated cyanide group of B_12_. The indicative signal of the vinyl stretching vibration at 1625 cm^−1^ of building block **2** is not anymore observed in the spectrum of the B_12_‐nanogel, underscoring the covalent incorporation of the targeting group into the gel during radical polymerization.

Below 1400 cm^−1^, the spectrum of the B_12_‐nanogel appears to be a superposition of **2** and the control nanogel. Signals at 1085 and 1000 cm^−1^ are assigned to the P─O stretch of the phosphodiester bond and the C─O─C stretching vibrations of the PEG‐ether moieties of the B_12_ targeting group, respectively [63, 64]. Two indicative signals between 1500 and 1700 cm^−1^ are observed for the nanogel as well. The first one is related to C═O stretching vibrations at 1635 cm^−1^, while the second corresponds to N─H bending at 1550 cm^−1^ of (NIPA) subunits of the control nanogel [65]. This region is significantly richer in signals for the 5′‐B_12_‐monomer **2**, where signals are assigned to the stretching of the C═O moieties of propionamide and the 5‐amide group [66] as well as N─H, and C─H bending vibrations of the corrin ring [67].

The spectrum of the B_12_‐nanogel also shows a broad signal at 1647 cm^−1^ and three signals at 1576, 1545, and 1499 cm^−1^. Whereas the latter three are slightly shifted compared to the control nanogel, the broad signal results from overlapping signals at 1667 and 1635 cm^−1^ of the control nanogel and **2**, respectively. Overall, all FTIR data, and especially the lacking signal of the vinyl group (─C═C─ bond) around 1625 cm^−1^ of the B_12_‐nanogel, suggest chemical incorporation of the B_12_‐targeting group into the nanogel polymer network.

Electron microscopy confirms uniform and spherical morphology (Figures 3a and S13), while EDS mapping shows cobalt distribution within the B_12_‐nanogel **3**, indicating incorporation of the B_12_‐monomer **2** into the polymer (Figure 3b). The mean particle sizes of approximately 50 nm were determined independently by SEM and TEM.

**FIGURE 3:**
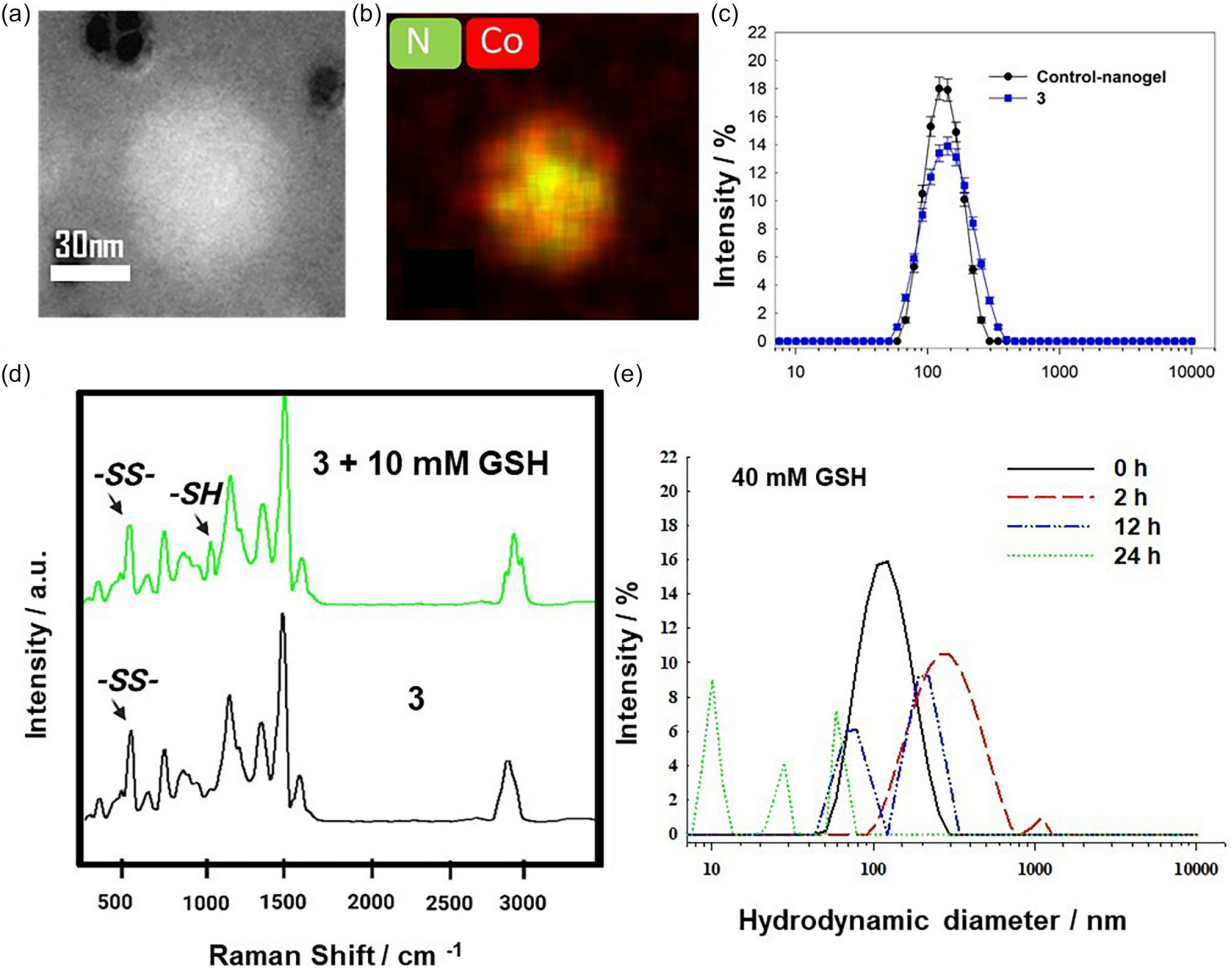
(a) TEM image of B_12_‐nanogel **3**. (b) EDS elemental mapping showing cobalt distribution within B_12_‐nanogel **3**. (c) Hydrodynamic diameter of nanogels determined by DLS. (d) Raman spectra of B_12_‐nanogel **3** treated with GSH (10 mM), recorded after 24 h. (e) Dynamic light scattering analysis of B_12_‐nanogel **3** treated with GSH (40 mM) between 0 and 24 h.

The nanogels form spherical particles with hydrodynamic diameters of 102 and 119 nm and polydispersity indices (PDI) of 0.11 and 0.23 for the control‐ and B_12_‐nanogel **3**, as determined by DLS (Figures 3c and S14 and Table S1).

The control nanogel showed a sharp reduction in hydrodynamic diameter around 35 °C (Figure S15a) comparable to the volume phase transition temperature (VPTT) of related NIPA‐containing micro/nanogels based [68]. The VPTT of the B_12_‐nanogel increased by 5 °C (i.e., 40 °C; Figure S15a), beyond which the microgel collapsed due to dehydration of the polymer network. This shrinkage is, however, less pronounced than for the control nanogel. This difference in thermoresponsive behavior is most likely caused by the B_12_‐targeting group, which is well‐known for forming hydrogen bonds with water molecules [69]. These additional hydrogen‐bonding interactions are expected to stabilize the hydrated state of the nanogels and delay the dehydration process, which nicely explains the increase of VPTT.

The pH sensitivity of the nanogels arises mainly from the presence of negatively charged AA units (Figure S15b–d). The zeta potential is a measure of the surface charge of particles and is crucial for understanding the stability and interaction potential of colloidal systems [70]. The dependence of the zeta potential of nanogels as a function of pH is depicted in Figure S15b. Both nanogels show a decrease in zeta potential from approximately 0 to –20 mV as the pH increases from 2 to 12. A pronounced transition to lower zeta potentials is observed around pH 4.5, corresponding to the p*K*_a_ value of the carboxylic acid functionality. Deprotonation of the carboxylic acid moieties of the gel causes a significant increase in surface charge, which explains the observed behavior. The B_12_‐targeting group shifts only slightly the zeta potential of the nanogel to more negative values. The effect of pH on the size and swelling (size) behavior of nanogels was assessed at different temperatures (20–70 °C) and pH values (pH 2.4, 5.3, and 11.8) (Figure S15c,d). The different pH values were carefully selected, as they reflect protonation (pH 2.4), partial (pH 5.3), and complete deprotonation (pH 11.8) of the carboxylic acid moieties. Aggregation of both gels was observed at a pH of 2.4 and above 30 °C due to the lack of repulsing carboxylate groups in the nanogel. At higher pH values, the carboxylic groups are ionized, resulting in an increased electrostatic repulsion and a swelling of the nanogel [71].

The presence of disulfide cross‐linkers from the BAC subunit in the nanogels enables redox responsiveness. The GSH‐triggered thiol‐disulfide exchange reaction occurs in two steps, which can lead to the degradation of the nanogel into smaller fragments. The mechanism is illustrated as follows:

R−S−S−R′ + GSH → R−S−S−G + R′−SH

R−S−S−G + GSH → R−SH + R′−SH + GS−SG

where *R* and *R*′ are parts of the nanogel polymer network, and GS−SG is glutathione disulfide (i.e., oxidized glutathione).

Exposure to glutathione (10 mM) induces analytical and structural changes consistent with cleavage of disulfide bonds and network degradation [72]. Intracellular levels in cancer cells can reach up to ~40 mM [73]. The strong signal at 518 cm^−1^ of the disulfide‐linker (─SS─) disappeared, and a new signal at 1028 cm^−1^ suggested formation of thiol bonds (─SH) (Figure ‍3d), whereas DLS measurements within 24 h suggested GSH‐triggered degradation of B_12_‐nanogel **3** into smaller fragments (Figure 3e).

Then, the nanogels were evaluated as carriers for doxorubicin. DOX exhibits a secondary amino group that is protonated to an ammonium functionality at physiological pH. The positively charged DOX is electrostatically attracted to the negatively charged carboxylate groups of the polymeric network during loadings at a pH of 7.4 (Scheme 1b). The UV–vis absorption spectrum confirmed the successful loading of DOX into the nanogel (Figure ‍S16). Quantitative drug loading and release were determined as detailed in Section 2.4 of the Supporting Information using the corresponding DOX calibration curves at pH 7.4 (HEPES buffer, 0.1 M) and pH 5 (acetate buffer, 0.1 M) (Figure S17). The drug loading capacity (DLC) and the drug encapsulation efficiency (DEE) were comparable for the control nanogel and B_12_‐nanogel **3**, respectively (control nanogel: DLC = 11%; DEE = 78%; and **3**: DLC = 10%; DEE = 70%).

Drug release from DOX@B_12_‐nanogel **4** was strongly dependent on pH and the presence of GSH (Figure 4a). Under physiological conditions (pH 7.4; HEPES buffer, 0.1 M) and in the absence of GSH, drug release remained minimal, with less than 16% released within the first 4 h. Subsequently, a plateau formed at approximately 18%. At a more acidic pH of 5.0 (acetate buffer, 0.1 M), drug release increased to 50%, reaching a plateau after the first 4 h. In the presence of biomimetic tumor concentrations of GSH (40 mM) at pH 5.0, DOX release was further enhanced by approx. 20% within the first 4 h, eventually reaching a release of as high as 75%. The release profile under these conditions shifted from an early plateau to a more gradual, sustained release phase, consistent with nanogel degradation via GSH‐mediated disulfide bond cleavage. These results highlight the combined effect of pH and GSH levels mimicking the intracellular tumor environment on promoting DOX release from DOX@B_12_‐nanogel **4**.

**FIGURE 4:**
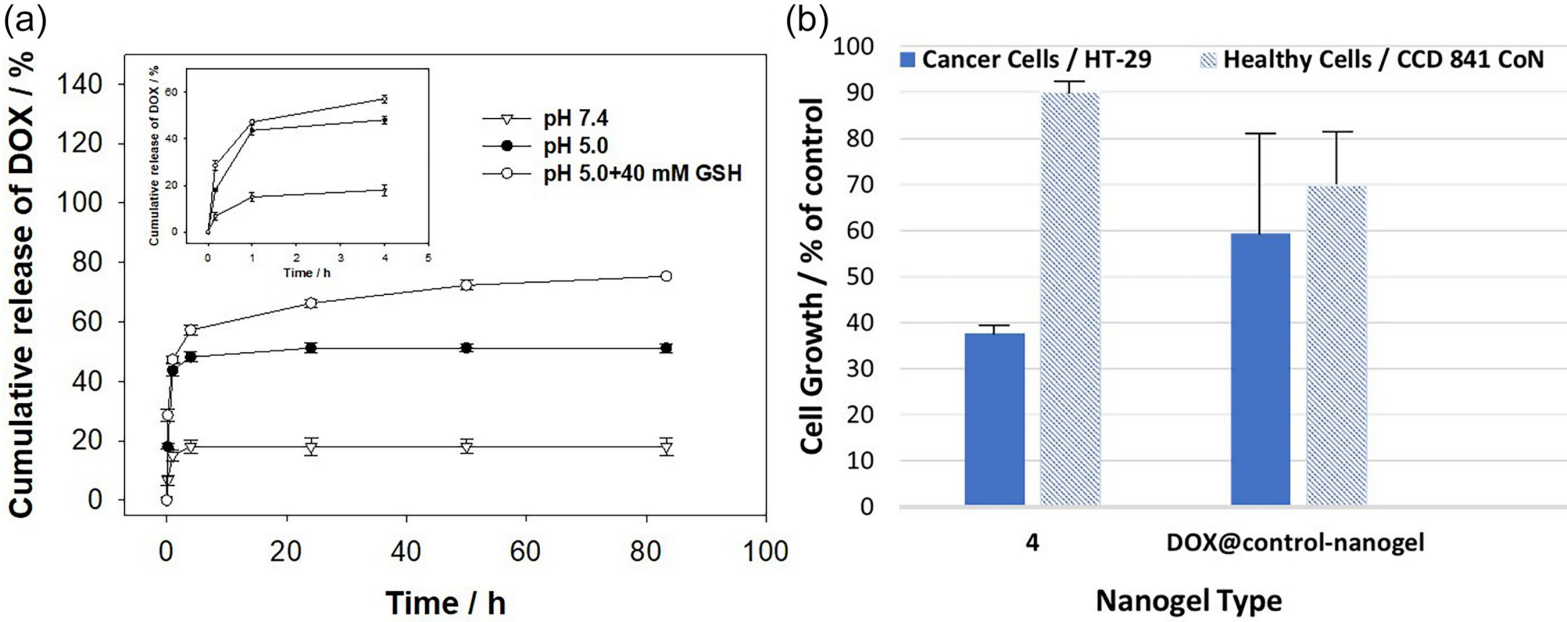
(a) Doxorubicin release profiles from DOX@B_12_‐nanogel **4** at pH 7.4, pH 5.0 in the absence of GSH, and pH 5.0 with additional GSH (40 mM). The inset shows the release profiles during the first 4 h. (b) Cytotoxicity of DOX@B_12_‐nanogel **4** (1 µM) toward cancer (HT‐29) and healthy (CCD 841 CoN) cell lines compared to the same experiments with the DOX@B_12_‐control gel (1 µM).

In addition, the B_12_‐nanogels **3** and **4** (i.e., before and after DOX loading) showed good stability in fetal bovine serum (FBS) over 72 h and at different temperatures (4, 25, and 37 °C) for over 20 days (Figure S18).

Proof‐of‐concept cytotoxicity studies showed that DOX@B_12_‐nanogel **4** maintains antiproliferative activity toward HT‐29 colorectal cancer cells (IC_50_ = 0.878 ± 0.102 µM), while exhibiting reduced toxicity toward healthy CCD 841 CoN cells (~90% viability at 1 µM DOX; IC_50_ ~ 30 µM). In comparison, the nonfunctionalized DOX@control nanogel showed higher toxicity toward CCD 841 CoN cells (~65% viability at 1 µM DOX; IC_50_ ~ 20 µM), indicating improved discrimination between cancer and healthy colorectal cells following B_12_ functionalization (Figures 4b and S19). Figure 4b presents a direct comparison of cell viability at a fixed DOX concentration of 1 µM, while the complete concentration‐dependent cytotoxicity data over the tested range of 0.1–10 µM are presented in Figure S19 and were used for IC_50_ determination.

These trends are consistent with a B_12_‐related contribution to cellular interactions, although TCII‐mediated uptake was not directly assessed.

## 3 Conclusion

In this work, we report the synthesis of a 5′‐modified vitamin B_12_ derivative bearing a polymerizable acrylamide moiety and its covalent incorporation into pH‐ and redox‐responsive nanogels. The resulting spherical B_12_ **‐**nanogels efficiently encapsulate the anticancer drug doxorubicin through electrostatic interactions between the cationic drug and the anionic nanogel network. Stimuli‐responsive release of doxorubicin was demonstrated under acidic conditions (pH 5) and in the presence of elevated glutathione concentrations (40 mM), mimicking the intracellular tumor microenvironment. In vitro studies further revealed that DOX@B_12_‐nanogels retain potent antiproliferative activity against HT‐29 colorectal cancer cells (IC_50_ = 0.878 ± 0.102 μM), while markedly reducing cytotoxicity toward healthy CCD 841 CoN cells compared with non‐B_12_‐functionalized control nanogels. Collectively, these findings highlight the potential of covalently engineered vitamin B_12_‐nanogels as a versatile platform for tumor‐targeted and stimuli‐responsive anticancer drug delivery.

## Funding

This work was supported by the Excellence Initiative – Research University, Poland, under grant number BOB‐IDUB‐622‐705/2023).

## Conflicts of Interest

The authors declare that a patent application related to this work has been filed (PCT/EP2026/051938).

## Supporting information

Supplementary Material

## Data Availability

The data that support the findings of this study are available in the Supporting Information of this article and in the UW Research Data Repository (Dane Badawcze UW) at https://doi.org/10.58132/JU8MZF.

## References

[1] National Cancer Institute , “Types of Cancer Treatment,” accessed August 23, 2022, https://www.cancer.gov/about‐cancer/treatment/types.

[2] American Cancer Society , “Understanding Cancer Treatment Options,” https://www.cancer.org/cancer/cancer‐basics/how‐cancer‐is‐treated.html.

[3] Brianna and S. H. Lee , “Chemotherapy: How to Reduce Its Adverse Effects While Maintaining the Potency?,” Medical Oncology 40 (2023): 88, 10.1007/s12032-023-01954-6. 36735206

[4] C. Zylberberg and S. Matosevic , “Pharmaceutical Liposomal Drug Delivery: A Review of New Delivery Systems and a Look at the Regulatory Landscape,” Drug Delivery 23, no. 9 (2016): 3319–3329, 10.3109/10717544.2016.1168493 27145899

[5] M. Cagel , F. C. Tesan , E. Bernabeu , et al., “Polymeric Mixed Micelles as Nanomedicines: Achievements and Perspectives,” European Journal of Pharmaceutics and Biopharmaceutics 113 (2017): 211–228, 10.1016/j.ejpb.2016.12.019. 28087380

[6] J. Ma , L. Fang , Z. Sun , et al., “Folate‐PEG‐PROTAC Micelles for Enhancing Tumor‐Specific Targeting Proteolysis In Vivo,” Advanced Healthcare Materials 13, no. 20 (2024): e2400109, 10.1002/adhm.202400109. 38676445

[7] Y. Patil , H. Shmeeda , Y. Amitay , P. Ohana , S. Kumar , and A. Gabizon , “Targeting of Folate‐Conjugated Liposomes With Co‐Entrapped Drugs to Prostate Cancer Cells via Prostate‐Specific Membrane Antigen (PSMA),” Nanomedicine: Nanotechnology, Biology and Medicine 14, no. 4 (2018): 1407–1416, 10.1016/j.nano.2018.04.011. 29680672

[8] T. Zhang , R. Yang , S. Yang , et al., “Research Progress of Self‐Assembled Nanogel and Hybrid Hydrogel Systems Based on Pullulan Derivatives,” Drug Delivery 25, no. 1 (2018): 278–292, 10.1080/10717544.2018.1425776. PMC6058595 29334800

[9] T. Wang , J. Ding , Z. Chen , et al., “Injectable, Adhesive Albumin Nanoparticle‐Incorporated Hydrogel for Sustained Localized Drug Delivery and Efficient Tumor Treatment,” ACS Applied Materials & Interfaces 16, no. 8 (2024): 9868–9879, 10.1021/acsami.3c18306. 38349713

[10] J. Shi , P. W. Kantoff , R. Wooster , and O. C. Farokhzad , “Cancer Nanomedicine: Progress, Challenges and Opportunities,” Nature Reviews Cancer 17, no. 1 (2017): 20–37, 10.1038/nrc.2016.108. PMC5575742 27834398

[11] D. Fan , Y. Cao , M. Cao , Y. Wang , Y. Cao , and T. Gong , “Nanomedicine in Cancer Therapy,” Signal Transduction and Targeted Therapy 8, no. 1 (2023): 293, 10.1038/s41392-023-01536-y. PMC10404590 37544972

[12] T. R. Hoare and D. S. Kohane , “Hydrogels in Drug Delivery: Progress and Challenges,” Polymer 49, no. 8 (2008): 1993–2007, 10.1016/j.polymer.2008.01.027.

[13] R. Langer and M. Chasin , Biodegradable Polymers as Drug Delivery Systems (Marcel Dekker, Inc, 1990).

[14] N. Kaushik , S. B. Borkar , S. K. Nandanwar , P. K. Panda , E. H. Choi , and N. K. Kaushik , “Nanocarrier Cancer Therapeutics With Functional Stimuli‐Responsive Mechanisms,” Journal of Nanobiotechnology 20 (2022): 152, 10.1186/s12951-022-01364-2. PMC8944113 35331246

[15] A. Garg , K. Shah , C. S. Chauhan , and R. Agrawal , “Ingenious Nanoscale Medication Delivery System: Nanogel,” Journal of Drug Delivery Science and Technology 92 (2024): 105289, 10.1016/j.jddst.2023.105289.

[16] M. Mackiewicz , S. Dagdelen , E. Walęka‐Bargieł , and M. Karbarz , “A Polyampholyte Core‐Shell Microgel as an Environmentally Sensitive Drug Carrier,” Arabian Journal of Chemistry 17 (2024): 105464, 10.1016/j.arabjc.2023.105464.

[17] N. Yang , X. Pan , X. Zhou , et al., “Biomimetic Nanoarchitectonics With Chitosan Nanogels for Collaborative Induction of Ferroptosis and Anticancer Immunity for Cancer Therapy,” Advanced Healthcare Materials 13, no. 7 (2024): 2302752, 10.1002/adhm.202302752. 37975280

[18] G. Cavallaro , G. Lazzara , S. Milioto , et al., “Nanohydrogel Formation Within the Halloysite Lumen for Triggered and Sustained Release,” ACS Applied Materials & Interfaces 10, no. 9 (2018): 8265–8273, 10.1021/acsami.7b19361. 29430922

[19] M. R. Rajeev , G. S. Lekshmi , R. Suriya , A. Lekshman , A. A. Rauf , and T. S. Anirudhan , “Transdermal Route of Administration of Multi‐Drugs Using a Metal Crosslinked Nanogel Device for Melanoma Therapy,” Chemical Engineering Journal 516 (2025): 164125, 10.1016/j.cej.2025.164125.

[20] R. de la Rica , D. Aili , and M. M. Stevens , “Enzyme‐Responsive Nanoparticles for Drug Release and Diagnostics,” Advanced Drug Delivery Reviews 64, no. 11 (2012): 967–978, 10.1016/j.addr.2012.01.002. 22266127

[21] X. M. Huang , Z. J. Luo , J. Guo , Q. J. Ruan , J. M. Wang , and X. Q. Yang , “Enzyme‐Adsorbed Chitosan Nanogel Particles as Edible Pickering Interfacial Biocatalysts and Lipase‐Responsive Phase Inversion of Emulsions,” Journal of Agricultural and Food Chemistry 68, no. 33 (2020): 8890–8899, 10.1021/acs.jafc.0c00116. 32687343

[22] S. Binauld and M. H. Stenzel , “Acid‐Degradable Polymers for Drug Delivery: A Decade of Innovation,” Chemical Communications 49, no. 21 (2013): 2082–2102, 10.1039/c2cc36589h. 23320254

[23] Y. Hu , M. Zhao , H. Wang , et al., “Exosome‐Sheathed ROS‐Responsive Nanogel to Improve Targeted Therapy in Perimenopausal Depression,” Journal of Nanobiotechnology 21 (2023): 261, 10.1186/s12951-023-02113-0. PMC10408189 37553718

[24] Y. Zhang , P. Dosta , J. Conde , N. Oliva , M. Wang , and N. Artzi , “Prolonged Local In Vivo Delivery of Stimuli‐Responsive Nanogels That Rapidly Release Doxorubicin in Triple‐Negative Breast Cancer Cells,” Advanced Healthcare Materials 9, no. 4 (2020): 1901101, 10.1002/adhm.201901101. 31957227

[25] X. Xu , X. Shi , B. Wang , et al., “Facilely Construct of GSH‐Responsive Nanogel by Photocrosslinking Based on a New Multi‐Functional Emulsify‐Crosslinking Agent for Comprehensively Improved Utilization Rate of Pesticides,” Chemical Engineering Journal 485 (2024): 150061, 10.1016/j.cej.2023.150061.

[26] E. Sameiyan , E. Bagheri , S. Dehghani , et al., “Aptamer‐Based ATP‐Responsive Delivery Systems for Cancer Diagnosis and Treatment,” Acta Biomaterialia 123 (2021): 110–122, 10.1016/j.actbio.2020.12.057. 33453405

[27] M. Mackiewicz , S. Dagdelen , M. S. Abubakar , J. Romanski , E. Waleka‐Bargiel , and M. Karbarz , “Stimuli‐Sensitive and Degradable Capsules as Drug Carriers With Decreased Toxicity Against Healthy Cells,” Polymer Degradation and Stability 212 (2023): 110349, 10.1016/j.polymdegradstab.2023.110349.

[28] J. T. Peters , S. S. Hutchinson , N. Lizana , I. Verma , and N. A. Peppas , “Synthesis and Characterization of Poly(N‐Isopropyl Methacrylamide) Core/Shell Nanogels for Controlled Release of Chemotherapeutics,” Chemical Engineering Journal 340 (2018): 58–65, 10.1016/j.cej.2018.01.009.

[29] S. Dagdelen , M. Mackiewicz , M. Osial , et al., “Redox‐Responsive Degradable Microgel Modified With Superparamagnetic Nanoparticles Exhibiting Controlled, Hyperthermia‐Enhanced Drug Release,” Journal of Materials Science 58 (2023): 4094–4114, 10.1007/s10853-023-08168-1.

[30] A. Seyfoori , S. A. Seyyed Ebrahimi , E. Samiei , and M. Akbari , “Multifunctional Hybrid Magnetic Microgel Synthesis for Immune‐Based Isolation and Post‐Isolation Culture of Tumor Cells,” ACS Applied Materials & Interfaces 11, no. 28 (2019): 24945–24958, 10.1021/acsami.9b02959. 31268286

[31] Z. Jin , Y. Wen , Y. Hu , et al., “MRI‐Guided and Ultrasound‐Triggered Release of NO by Advanced Nanomedicine,” Nanoscale 9, no. 10 (2017): 3637–3645, 10.1039/C7NR00231A. 28247895

[32] X. Ai , J. Mu , and B. Xing , “Recent Advances of Light‐Mediated Theranostics,” Theranostics 6, no. 13 (2016): 2439–2457, 10.7150/thno.16088. PMC5118606 27877246

[33] C. Zhang , W. Sun , Y. Wang , et al., “Gd‐/CuS‐Loaded Functional Nanogels for MR/PA Imaging‐Guided Tumor‐Targeted Photothermal Therapy,” ACS Applied Materials & Interfaces 12, no. 8 (2020): 9107–9117, 10.1021/acsami.9b23413. 32003962

[34] X. Jia , X. Zhao , K. Tian , et al., “Novel Fluorescent pH/Reduction Dual Stimuli‐Responsive Polymeric Nanoparticles for Intracellular Triggered Anticancer Drug Release,” Chemical Engineering Journal 295 (2016): 468–493, 10.1016/j.cej.2016.03.040.

[35] D. Huang , H. Qian , H. Qiao , W. Chen , J. Feijen , and Z. Zhong , “Bioresponsive Functional Nanogels as an Emerging Platform for Cancer Therapy,” Expert Opinion on Drug Delivery 15, no. 7 (2018): 703–716, 10.1080/17425247.2018.1497607. 29976103

[36] F. Li , Z. Liang , and D. Ling , “Smart Organic‐Inorganic Nanogels for Activatable Theranostics,” Current Medicinal Chemistry 26, no. 8 (2019): 1366–1376, 10.2174/0929867324666170920164614. 28933302

[37] S. Hajebi , N. Rabiee , M. Bagherzadeh , et al., “Stimulus‐Responsive Polymeric Nanogels as Smart Drug Delivery Systems,” Acta Biomaterialia 92 (2019): 1–18, 10.1016/j.actbio.2019.05.018. PMC6661071 31096042

[38] Y. Xiao , K. Pandey , A. Nicolás‐Boluda , et al., “Synergic Thermo‐ and pH‐Sensitive Hybrid Microgels Loaded With Fluorescent Dyes and Ultrasmall Gold Nanoparticles for Photoacoustic Imaging and Photothermal Therapy,” ACS Applied Materials & Interfaces 14, no. 49 (2022): 54439–54457, 10.1021/acsami.2c12796. 36468426

[39] J. W. Nichols and Y. H. Bae , “EPR: Evidence and Fallacy,” Journal of Controlled Release 190 (2014): 451–464, 10.1016/j.jconrel.2014.03.057. 24794900

[40] F. Danhier , “To Exploit the Tumor Microenvironment: Since the EPR Effect Fails in the Clinic, What Is the Future of Nanomedicine?,” Journal of Controlled Release 244 (2016): 108–121, 10.1016/j.jconrel.2016.11.015. 27871992

[41] S. Wilhelm , A. J. Tavares , Q. Dai , et al., “Analysis of Nanoparticle Delivery to Tumours,” Nature Reviews Materials 1, no. 5 (2016): 16014, 10.1038/natrevmats.2016.14.

[42] C. J. Cheng , G. T. Tietjen , J. K. Saucier‐Sawyer , and W. M. Saltzman , “A Holistic Approach to Targeting Disease With Polymeric Nanoparticles,” Nature Reviews Drug Discovery 14, no. 4 (2015): 239–247, 10.1038/nrd4503. PMC4451203 25598505

[43] N. V. Nukolova , H. S. Oberoi , S. M. Cohen , A. V. Kabanov , and T. K. Bronich , “Folate‐Decorated Nanogels for Targeted Therapy of Ovarian Cancer,” Biomaterials 32, no. 23 (2011): 5417–5426, 10.1016/j.biomaterials.2011.04.006. PMC3255291 21536326

[44] M. Narwade , A. Shaikh , K. R. Gajbhiye , P. Kesharwani , and V. Gajbhiye , “Advanced Cancer Targeting Using Aptamer Functionalized Nanocarriers for Site‐Specific Cargo Delivery,” Biomaterials Research 27 (2023): 42, 10.1186/s40824-023-00365-y. PMC10164340 37149607

[45] Y. J. Chae , K.‐G. Lee , D. Oh , S.‐K. Lee , Y. Park , and J. Kim , “Antibody‐Conjugated Nanogel With Two Immune Checkpoint Inhibitors for Enhanced Cancer Immunotherapy,” Advanced Healthcare Materials 13, no. 19 (2024): e2400235, 10.1002/adhm.202400235. 38569198

[46] C. Rodríguez‐Nava , C. Ortuño‐Pineda , B. Illades‐Aguiar , et al., “Mechanisms of Action and Limitations of Monoclonal Antibodies and Single Chain Fragment Variable (scFv) in the Treatment of Cancer,” Biomedicines 11, no. 6 (2023): 1610, 10.3390/biomedicines11061610. PMC10295864 37371712

[47] K. Patel and A. Sobczyńska‐Malefora , “The Adverse Effects of an Excessive Folic Acid Intake,” European Journal of Clinical Nutrition 71, no. 2 (2017): 159–163, 10.1038/ejcn.2016.194. 27731331

[48] R. Banerjee , C. Gherasim , and D. Padovani , “The Tinker, Tailor, Soldier in Intracellular B_12_ Trafficking,” Current Opinion in Chemical Biology 13, no. 4 (2009): 484–491, 10.1016/j.cbpa.2009.07.007. PMC5750051 19665918

[49] A. K. Petrus , T. J. Fairchild , and R. P. Doyle , “Traveling the Vitamin B_12_ Pathway: Oral Delivery of Protein and Peptide Drugs,” Angewandte Chemie, International Edition 48, no. 6 (2009): 1022–1028, 10.1002/anie.200800865. 19072807

[50] F. Zelder , “Recent Trends in the Development of Vitamin B_12_ Derivatives for Medicinal Applications,” Chemical Communications 51, no. 74 (2015): 14004–14017, 10.1039/C5CC05569G. 26287029

[51] R. Alberto and F. Zelder , “Coordination Chemistry of Corrinoids. in Vitamin B 12 for Radioimaging and as Carrier for Targeted Drug Delivery,” 25 (2012): 83–130, 10.1142/9789814397605_0018.

[52] A. N. W. Kuda‐Wedagedara , J. L. Workinger , E. Nexo , R. P. Doyle , and N. Viola‐Villegas , “ ^89^Zr‐Cobalamin PET Tracer: Synthesis, Cellular Uptake, and Use for Tumor Imaging,” ACS Omega 2, no. 10 (2017): 6314–6320, 10.1021/acsomega.7b01180. PMC5664145 29104950

[53] A. Jackowska , M. Chromiński , M. Giedyk , and D. Gryko , “5′‐Vitamin B_12_ Derivatives Suitable for Bioconjugation *via* the Amide Bond,” Organic & Biomolecular Chemistry 16, no. 6 (2018): 936–943, 10.1039/c7ob02898a. 29336455

[54] K. Zelenka , H. Brandl , B. Spingler , and F. Zelder , “Coordination Chemistry and Biological Activity of 5′‐OH Modified Quinoline–B_12_ Derivatives,” Dalton Transactions 40, no. 38 (2011): 9665–9667, 10.1039/c1dt11161b. 21850334

[55] P. M. Pathare , D. S. Wilbur , S. Heusser , E. V. Quadros , P. McLoughlin , and A. C. Morgan , “Synthesis of Cobalamin−Biotin Conjugates that Vary in the Position of Cobalamin Coupling. Evaluation of Cobalamin Derivative Binding to Transcobalamin II,” Bioconjugate Chemistry 7, no. 2 (1996): 217–232, 10.1021/bc9600022. 8983344

[56] D. A. Collins and H. P. C. Hogenkamp , “Transcobalamin II Receptor Imaging via Radiolabeled Diethylene‐Triaminepentaacetate Cobalamin Analogs,” Journal of Nuclear Medicine 38, no. 5 (1997): 717–723. 9170435

[57] M. Lee and C. B. Grissom , “Design, Synthesis, and Characterization of Fluorescent Cobalamin Analogues With High Quantum Efficiencies,” Organic Letters 11, no. 12 (2009): 2499–2502, 10.1021/ol900401z. 19441855

[58] N. Thepphankulngarm , P. Wonganan , C. Sapcharoenkun , T. Tuntulani , and P. Leeladee , “Combining Vitamin B_12_ and Cisplatin‐Loaded Porous Silica Nanoparticles via Coordination: A Facile Approach to Prepare a Targeted Drug Delivery System,” New Journal of Chemistry 41 (2017): 13823–13829, 10.1039/C7NJ02754K.

[59] Y. Yoshimura , T. Iwahashi , T. Kasuya , et al., “Methylcobalamin‐Containing Nanofiber Sheets Have Better Neuroprotective Effects Than Small Intestinal Submucosa Sheets,” Scientific Reports 15 (2025): 950, 10.1038/s41598-024-78933-y. PMC11704040 39762256

[60] Y. Yu , Y. G. Peng , Y. J. Zhong , et al., “Innovative pH‐Responsive Chitosan Hydrogel: A Systematic Study on VB_12_ Controlled Release and Multifunctional Biological Effects via Synergistic Grafting of Cysteine and Arginine,” International Journal of Biological Macromolecules 322, Part 3 (2025): 146718, 10.1016/j.ijbiomac.2025.143628. 40784396

[61] Supporting Information.

[62] K. Nakamoto , Infrared and Raman Spectra of Inorganic and Coordination Compounds: Part A: Theory and Applications in Inorganic Chemistry (Wiley, 2008).

[63] G. Socrates , Infrared and Raman Characteristic Group Frequencies: Tables and Charts, 3rd ed. (Wiley, 2001).

[64] N. B. Colthup , L. H. Daly , and S. E. Wiberley , Introduction to Infrared and Raman Spectroscopy, 3rd ed. (Academic Press, 1990).

[65] B. Smith , Infrared Spectral Interpretation: A Systematic Approach (CRC Press, 1998).

[66] L. J. Bellamy , The Infrared Spectra of Complex Molecules (Springer, 1980).

[67] K. N. Aziz , K. M. Ahmed , R. A. Omer , A. F. Qader , and E. I. Abdulkareem , “A Review of Coordination Compounds: Structure, Stability, and Applications,” Reviews in Inorganic Chemistry 45, no. 1 (2025): 1–19, 10.1515/revic-2024-0035.

[68] D. K. Gupta , D. Karthickeyan , B. V. R. Tata , and T. R. Ravindran , “Temperature‐Driven Volume Phase Transition of a Single Stimuli‐Responsive Microgel Particle Using Optical Tweezers,” Colloid and Polymer Science 294, no. 12 (2016): 1901–1908, 10.1007/s00396-016-3952-1

[69] M. Pratt , “Chemistry and Biochemistry of B12,” ed. R. Banerjee , 1st ed. (Wiley‐Interscience, 1999).

[70] R. J. Hunter , Zeta Potential in Colloid Science: Principles and Applications (Academic Press, 1981).

[71] M. Wiśniewska , T. Urban , E. Grządka , V. I. Zarko , and V. M. Gun’ko , “Comparison of Adsorption Affinity of Polyacrylic Acid for Surfaces of Mixed Silica‐Alumina,” Colloid and Polymer Science 292, no. 3 (2014): 699–705, 10.1007/s00396-013-3103-x. PMC3940901 24610970

[72] R. Dabas and N. Kamaly , “Redox‐Responsive Nanogels for Precision Protein Delivery,” European Polymer Journal 215 (2024): 113183, 10.1016/j.eurpolymj.2024.113183.

[73] M. P. Gamcsik , M. S. Kasibhatla , S. D. Teeter , and O. M. Colvin , “Glutathione Levels in Human Tumors,” Biomarkers 17, no. 8 (2012): 671–691, 10.3109/1354750X.2012.715672. PMC3608468 22900535

