# Supplementary material for: Vitamin B12‐Functionalized Nanogels for pH‐, and Redox‐Responsive Doxorubicin Delivery
Source: Chembiochem. 2026 Oct 26;27(20):e70580. doi: 10.1002/cbic.70580 (PMC13698639; doi:10.1002/cbic.70580)
Supplement: Supplementary file 1 [file CBIC-27-e70580-s001.pdf]

Vitamin B_{12}-functionalized nanogels for pH-, and redoxresponsive doxorubicin delivery

Serife Dagdelen[a,b], Paula D. Mestizo[c], Marcin Mackiewicz[d], Agnieszka Kurdyn[e], Ewa Augustin[e], Marcin Karbarz[a,b*] and Felix Zelder[c*]

[a] *University of Warsaw, Faculty of Chemistry, Pasteura 1, 02-093 Warsaw, Poland*

[b] *University of Warsaw, Biological and Chemical Research Center, Żwirki i Wigury 101, 02-089, Warsaw,*

Poland

[c] *Department of Chemistry, University of Zürich, Winterthurerstrasse 190, 8057 Zürich, Switzerland* [d]

[d] *Department of Molecular Biology, Institute of Biochemistry, Faculty of Biology, University of Warsaw,*

Miecznikowa 1, Warsaw, 02-096, Poland

[e] *Department of Pharmaceutical Technology and Biochemistry, Faculty of Chemistry, Gdańsk University of Technology, Narutowicza 11/12, 80-233 Gdańsk, Poland*

*Corresponding authors

M. K. karbarz@chem.uw.edu.pl

F. Z. felix.zelder@chem.uzh.ch

Contents

2. Materials and methods 3
  2.1. General 3
  2.2 Instrumentation 4
  2.3 Other measurements 5
  2.4 Drug loading and release 5
  2.5 Cell studies 5
4. Synthesis 9
  4.1 Synthesis of 5’-B_{12}-PEG_3^+ trifluoroacetate 9
  4.2 Synthesis of B_{12} monomer 2 10
  4.3 Synthesis of B_{12}-nanogel 3 11
  4.4 Synthesis of control gel 12
5. Analytical Data 13
  5.1 Chromatograms and Optical spectra 13
  5.2 NMR spectra, table of NMR data and discussion 16
  5.3 Nanogel Size and Morphology 20
  5.4 Cytotoxicity data 25
References 25

2. Materials and methods

2.1. General

Chemicals and reagents.

All reagents were of analytical grade and used as received unless otherwise stated. *N*isopropylacrylamide (NIPA), ammonium persulfate (APS), acrylic acid (AA), *N,N'*bis(acryloyl)cystamine (BAC), *L*-glutathione (GSH), vitamin B_{12} (CNCbl), 4,7,10-trioxa1,13-tridecanediamine, and HEPES buffer were obtained from Sigma–Aldrich. NaOH, HCl_{aq}, and *N,N,N′,N′*-tetramethylethylenediamine (TEMED) were purchased from POCH. Doxorubicin hydrochloride (DOX) was obtained from LC Laboratories. Dulbecco's phosphate-buffered saline (D-PBS) was purchased from VWR. Fetal bovine serum (FBS) was obtained from ScienCell or Biowest.

NIPA was recrystallized twice from benzene/hexane (9:1, v/v) prior to use.

Solvents.

Solvents were of analytical, HPLC, or LC–MS grade. Ultrapure water was obtained from a Milli-Q or Hydrolab system.

General procedures.

Reactions were monitored by LC–MS. Solvents were removed under reduced pressure using a rotary evaporator. Solid-phase extraction (SPE) was performed using RP-18 cartridges (MeOH/H_2O conditioning). Products were isolated by centrifugation and dried under high vacuum (≤8 × 10^{-2} mbar) overnight.

2.2 Instrumentation

Preparative HPLC.

Purification was performed on a Shimadzu Nexera Prep HPLC system equipped with UV detection (280 nm) using a Nucleosil column (250 × 21 mm). Elution was carried out with CH_3CN/H_2O (0.1% CF_3COOH) at 40 mL min^{-1} using a gradient of 20–95% CH_3CN.

LC–MS.

Measurements were performed on a Vanquish™ UHPLC system coupled to an ISQEM mass spectrometer (HESI source). Separation was achieved on a BEH C18 column (2.1 × 50 mm, 1.7 µm) at 40 °C using CH_3CN/H_2O (0.1% HCOOH) with gradient elution (5–100% CH_3CN, 0.6 mL min^{-1}).

HR-ESI-MS.

High-resolution spectra were recorded on a Q Exactive mass spectrometer with mass accuracy <2 ppm.

NMR spectroscopy.

1H and ^{13}C NMR spectra were recorded at 298 K on a Bruker AVANCE NEO 500 MHz spectrometer in DMSO-d_6, D_2O, or CD_3OD. Chemical shifts are reported in ppm relative to residual solvent signals.

UV–Vis spectroscopy.

UV–Vis absorption spectra were recorded from 200-800 nm using an Evolution 300 UV–Vis spectrophotometer (Thermo Scientific) in 1 cm quartz cuvettes at room temperature.

Dynamic light scattering (DLS).

Hydrodynamic diameters were measured using a Malvern Zetasizer Nano ZS (λ = 632.8 nm, 173° scattering angle). Samples were equilibrated for 5 min at the desired temperature before measurement. Measurements were performed in triplicate (n = 3), and results are reported as mean ± SD. Unless otherwise stated, measurements were performed at 37 °C.

FTIR spectroscopy.

FTIR spectra were recorded using an ATR-equipped Nicolet iS50 spectrometer.

Electron microscopy (SEM, TEM).

SEM images were obtained using a Zeiss Merlin microscope operated at 3 kV. A drop of nanogel dispersion was dried at room temperature and coated with a 5 nm Au-Pd layer prior to imaging. TEM images were obtained using a Zeiss Libra 1200 microscope after depositing the nanogel dispersion onto a Formvar-coated copper grid and drying at room temperature.

Raman spectroscopy.

Raman spectra were recorded using a LabRAM Aramis system (532 nm laser).

2.3 Other measurements

The pH of nanogel dispersions was adjusted using $NaOH_{aq}$ or HCl_{aq} and measured with a pH meter. Ionic strength was maintained at 0.01 M. For GSH-containing systems, pH was adjusted to ~5.0.

2.4 Drug loading and release

Doxorubicin loading was performed by thermal cycling. The control nanogel or B_{12}-nanogel **4** (3 mL) was mixed with HEPES buffer (3 mL, pH 7.4, 0.1 M) containing DOX (1.5 mg), heated to 42 °C for 10 min, and incubated at 25 °C for 24 h. Excess DOX was removed by dialysis (MWCO 10 kDa). The amount of free DOX was determined spectrophotometrically at 480 nm using a DOX calibration curve. The drug loading capacity (DLC) was assessed using the formula: $DLC\% = \frac{m_{total}^{DOX} - m_{free}^{DOX}}{m_{nanogel}} \times 100\%$, and the drug encapsulation efficiency (DEE) was assessed using the following equation: $DEE\% = \frac{m_{total}^{DOX} - m_{free}^{DOX}}{m_{total}^{DOX}} \times 100\%$.

Release studies were performed by dialysis at 37 °C in HEPES (pH 7.4, 0.1 M) or acetate buffer (pH 5.0, 0.1 M) in the absence or presence of GSH. At predetermined

time points, aliquots of the release medium were withdrawn and replaced with an equal volume of fresh buffer to maintain a constant release volume and sink conditions. Released DOX was quantified spectrophotometrically at 480 nm using the corresponding calibration curves prepared in HEPES and acetate buffers. Control nanogels were treated analogously.

2.5 Cell studies

Cell culture.

HT-29 human colon adenocarcinoma and CCD 841 CoN normal human colon epithelial cells (ATCC) were used. HT-29 cells were cultured in McCoy's 5A medium supplemented with 10% heat-inactivated FBS, 100 µg mL^{-1} streptomycin, and 100 U mL^{-1} penicillin. CCD 841 CoN cells were maintained in Minimum Essential Medium Eagle supplemented with 10% FBS. Cells were cultured at 37 °C in a humidified atmosphere containing 5% CO_2.

Cytotoxicity assay.

Cell viability was evaluated using the MTT assay. HT-29 and CCD 841 CoN cells were seeded in 96-well plates at densities of 2×10^3 and 7×10^3 cells/well, respectively. After 24 h preincubation, cells were treated with the tested compounds (≤60 µM) for 72 h. MTT (400 µg/well) was added for 2 h, followed by dissolution of the formazan crystals in DMSO. Absorbance was measured at 540 nm using an iMark™ microplate reader (Bio-Rad, USA), and IC_{50} values were determined from dose–response curves relative to untreated controls. Results were obtained from at least four independent experiments.

3. Reaction Schemes.

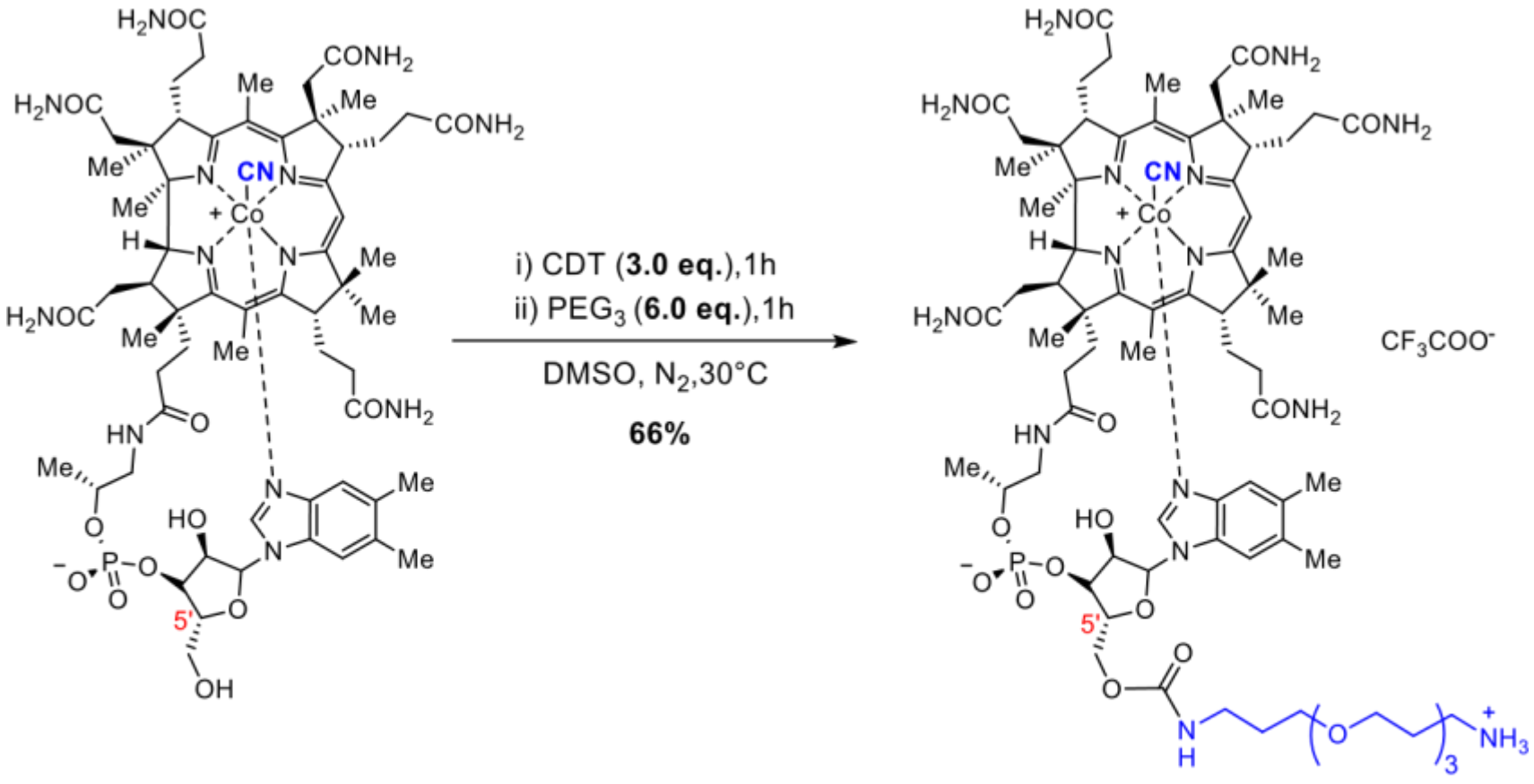

1

$C_{63}H_{88}CoN_{14}O_{14}P$
1355.39 g/mol

5'-B$_{12}$-PEG$_3$$^+$ trifluoroacetate

$C_{76}H_{111}CoF_3N_{16}O_{20}P$
1715.72 g/mol

Scheme S1. Synthesis of the precursor **5'-B$_{12}$-PEG$_3$$^+$ trifluoroacetate**.

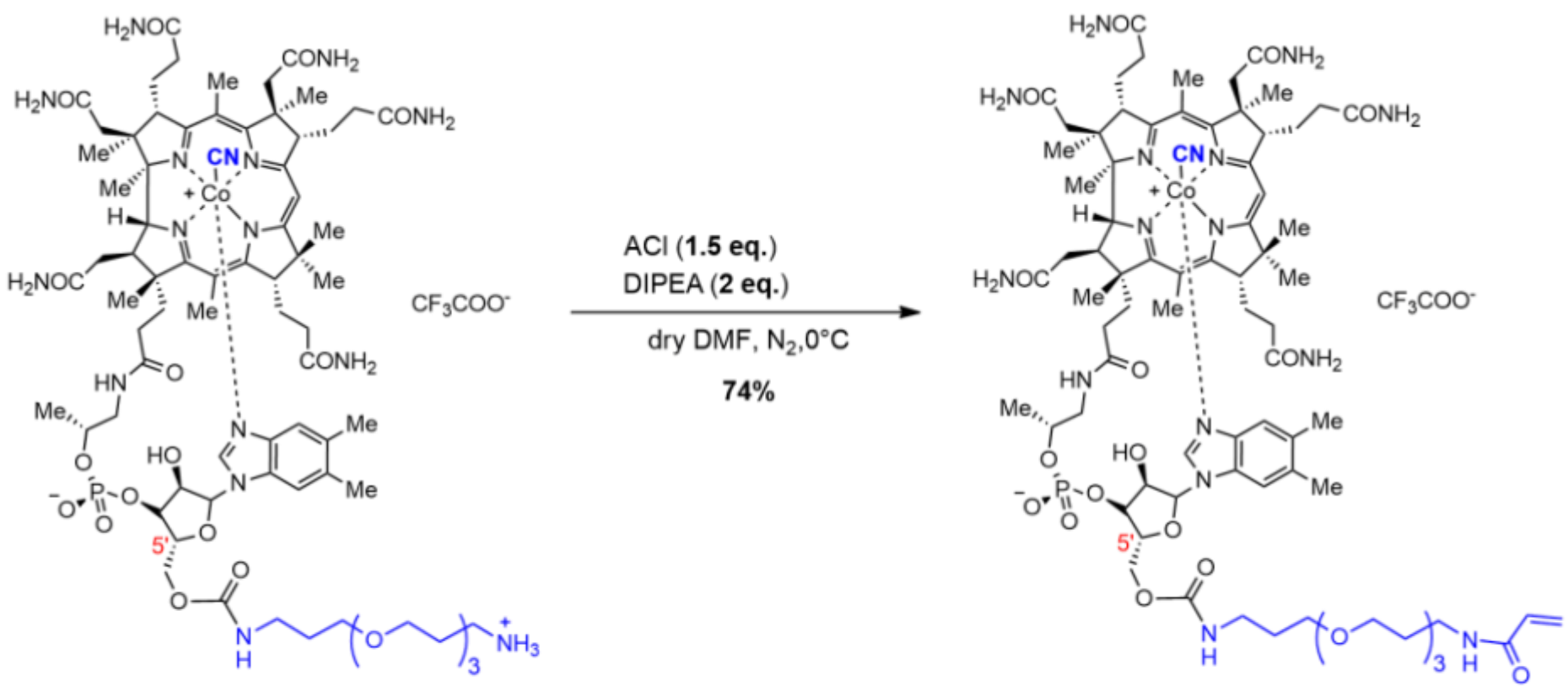

5'-B$_{12}$-PEG$_3$$^+$ trifluoroacetate

$C_{76}H_{111}CoF_3N_{16}O_{20}P$
1715.72 g/mol

2

$C_{79}H_{113}CoF_3N_{16}O_{21}P$
1769.74 g/mol

Scheme S2. Synthesis B$_{12}$-monomer **2**.

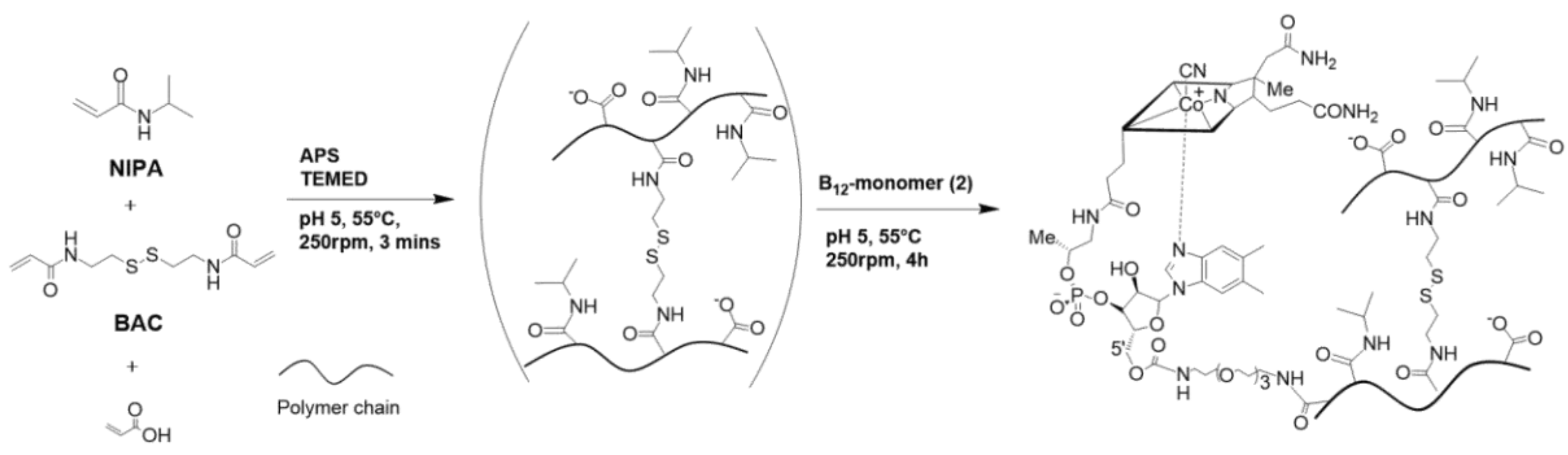

Scheme S3. Synthesis B$_{12}$-nanogel **3**.

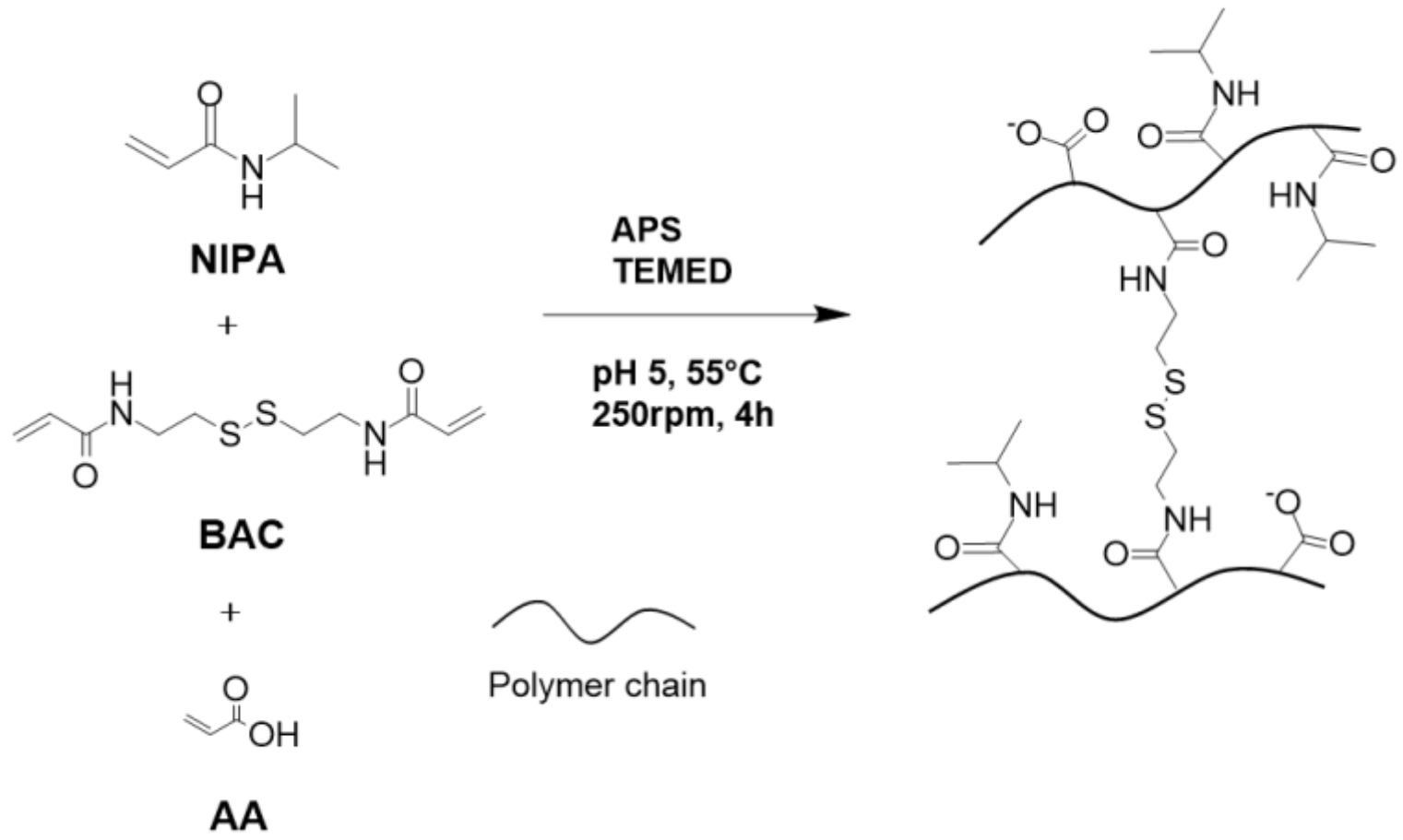

Scheme S4. Synthesis of **control-nanogel.**

4. Synthesis

4.1 Synthesis of 5’-B_{12}-PEG_3^+ trifluoroacetate

The synthesis of the **5’-B_{12}-PEG_3^+** precursor was slightly modified according to a reported procedure (Scheme S1).[1] In a Schlenk flask and under an inert $N_{2(g)}$ atmosphere, CNCbl (200.0 mg, 0.148 mmol, 1.0 eq.) was added to DMSO (1.5 mL) under stirring. After the solid was completely dissolved, CDT (72.8 mg, 0.442 mmol, 3.0 eq.) was added. The reaction mixture was then heated to 30 °C for 1h. The activation step was completed after 1h as indicated by reaction control using LCMS. Afterwards, 4,7,10-trioxa-1,13-tridecadiamine (192 µL, 0.88 mmol, 5.95 eq.) was added. The reaction mixture was left stirring for an additional 1h under the same temperature. The resulting crude mixture was precipitated over Et_2O: DCM 2:1 (10 mL), centrifuged and the DMSO rich supernatant discarded. The remaining crude was washed using DCM (2 mL x 2) and Et_2O (5 mL x 2). After drying the solid to remove all organic solvents, it was purified using preparative HPLC (method 1, t_{ret}= 11.2 min). CH_3CN was then evaporated from the collected pure fractions and the product passed through a SepPack C18 cartridge, washed with H_2O (20 mL) and finally eluted with MeOH (5mL). The final red solid was obtained after precipitation from a concentrated MeOH solution (200 µL final volume) using methyl tert-butyl ether (MTBE) (166.9 mg, 0.097 mmol, 66 %).

UV-Vis (H_2O, c = 4.0·10^{-5} M): (4.25), 519 (3.71), 549 (3.75). ***LC–MS*** (MS+) (t_{ret} = 1.73 min): 801.36 (100, $[M + 2H]^{2+}$, m/z_{calc}: 801.37), 1601.85 (20, $[M + H]^+$, m/z_{calc}: 1601.73 for $C_{74}H_{111}CoN_{16}O_{18}P^+$***).***

^{1}H NMR (DMSO-*d6*, c = 1.2· 10^{-2} M) δ 8.07 (s, 3H), 7.71 (d, J = 9.5 Hz, 2H), 7.64 (s, 1H), 7.56 (d, J = 16.0 Hz, 2H), 7.37 (s, 1H), 7.28 (s, 1H), 7.19 (s, 2H), 7.16 (s, 1H), 7.08 (s, 1H), 7.03 (s, 1H), 6.99 (s, 1H), 6.79 (s, 1H), 6.72 (s, 1H), 6.53 (s, 1H), 6.44 (s, 1H), 6.41 (s, 1H), 6.20 (s, 1H), 5.92 (s, 1H), 4.68 (d, J = 7.2 Hz, 1H), 4.63 (q, J = 7.7 Hz, 1H), 4.25 (d, J = 10.5 Hz, 1H), 4.20 – 4.11 (m, 2H), 4.09 (d, J = 7.4 Hz, 1H), 4.04 (s, 1H), 3.94 (d, J = 11.1 Hz, 1H), 3.75 – 3.67 (m, 1H), 3.58 – 3.45 (m, 12H), 3.45 – 3.38 (m, 2H), 2.99 – 2.90 (m, 1H), 2.84 (dd, J = 12.3, 6.4 Hz, 2H), 2.80 – 2.74 (m, 1H), 2.58 (d, J = 12.2 Hz, 1H), 2.48 (s, 3H), 2.44 (s, 2H), 2.38 (s, 3H), 2.37 – 2.30 (m, 3H), 2.26 (t, J = 14.6 Hz, 2H), 2.16 (d, J = 7.2 Hz, 8H), 2.08 – 1.96 (m, 3H), 1.79 (q, J = 7.1, 6.1 Hz, 5H), 1.73 (s, 2H), 1.69 (s, 3H), 1.63 (dt, J = 14.7, 6.9 Hz, 3H), 1.51 (dt, J = 15.1, 8.0 Hz, 1H), 1.32 (s, 3H), 1.23 (s, 3H), 1.18 (s, 3H), 1.07 (d, J = 7.2 Hz, 7H), 0.87 (s, 1H), 0.33 (s, 3H).

^{13}C NMR (DMSO-*d6*, *c* = 1.2· 10^{-2} M) δ 179.88, 178.48, 175.52, 174.49, 173.96, 173.94, 173.50, 173.04, 172.98, 171.74, 171.71, 165.60, 165.06, 156.58, 142.43, 136.60, 133.27, 131.87, 129.82, 116.67, 116.27, 111.52, 106.35, 103.34, 93.93, 86.33, 84.66, 79.67, 75.01, 73.11, 72.56, 71.52, 70.04, 69.96, 69.76, 69.74, 68.93, 68.04, 67.78, 63.08, 58.93, 55.34, 54.31, 53.35, 50.70, 49.06, 47.71, 46.89, 44.80, 42.36, 42.12, 38.39, 37.88, 37.21, 35.36, 34.17, 32.02, 31.94, 31.77, 31.66, 30.99, 30.58, 29.80, 27.47, 26.12, 25.91, 20.23, 20.20, 20.12, 20.09, 19.96, 19.00, 16.75, 16.71, 15.90, 15.30, 1.41.

4.2 Synthesis of B_{12} monomer 2

Under an inert $N_{2(g)}$ atmosphere, **5'-B_{12}-PEG_3^+ trifluoroacetate** (100.0 mg, 0.062 mmol, 1.0 eq.) was dissolved in DMF (2 mL) under stirring and diisopropylethylamine (DIPEA) (19.3 µL, 0.12 mmol, 2 eq.) was added. The reaction mixture was cooled to 0°C and after 30 min, acryloyl chloride (ACl) (7.6 µL, 0.094 mmol, 1.5 eq.) was added. The reaction was completed after 1h, as indicated by reaction control using LC-MS. The resulting crude mixture was precipitated over Et_2O: DCM 2:1 (15 mL), centrifuged and the DMF rich supernatant discarded. The remaining crude was washed using DCM (2 mL x 2) and Et_2O (5 mL x 2). After drying the solid to remove all organic solvents, it was purified using preparative HPLC (t_{ret}= 11.2 min). CH_3CN was then evaporated from the collected pure fractions and the product passed through a SepPack C18 cartridge, washed with H_2O (20 mL) and finally eluted with MeOH (5 mL). The final red solid was obtained after precipitation from a concentrated MeOH solution (200 µL final volume) using methyl *tert*-butyl ether (MTBE) (166.9 mg, 0.097 mmol, 94 %).

UV-Vis (H_2O, c = $4.0\cdot10^{-5}$ M): 280.4, 361.7, 519, 549. ***LC–MS** (MS$^+$)* (t_{ret} = 1.99 min): 828.53 (100, $[M + 2H]^{2+}$, m/z_{calc}: 828.37), 1655.82 (20, $[M + H]^+$, m/z_{calc}: 1655.74 for $C_{77}H_{112}CoN_{16}O_{19}P$).

^1H NMR (DMSO-d6, c = 1.1.10^{-2}M) δ 8.19 (t, J = 5.3 Hz, 1H), 7.77 (s, 1H), 7.72 (d, J = 19.0 Hz, 2H), 7.57 (d, J = 36.3 Hz, 2H), 7.36 (d, J = 7.4 Hz, 3H), 7.17 (d, J = 26.1 Hz, 2H), 7.08 (s, 1H), 7.01 (d, J = 13.0 Hz, 2H), 6.76 (d, J = 19.1 Hz, 2H), 6.53 (s, 1H), 6.43 (s, 1H), 6.37 (s, 1H), 6.28 – 6.17 (m, 2H), 6.05 (dd, J = 17.1, 2.2 Hz, 1H), 5.89 (s, 1H), 5.54 (dd, J = 10.2, 2.2 Hz, 1H), 4.67 (d, J = 7.4 Hz, 1H),

4.63 (s, 1H), 4.29 (d, J = 10.4 Hz, 1H), 4.19 – 4.11 (m, 1H), 4.08 (d, J = 7.6 Hz, 1H), 4.02 (dd, J = 11.2, 7.2 Hz, 2H), 3.93 (d, J = 10.8 Hz, 1H), 3.69 (dd, J = 9.9, 5.9 Hz, 1H), 3.51 (dt, J = 5.4, 3.1 Hz, 3H), 3.49 – 3.43 (m, 4H), 3.42 – 3.35 (m, 1H), 3.19 – 3.12 (m, 2H), 3.02 (q, J = 6.8, 6.3 Hz, 2H), 2.88 (dd, J = 13.9, 7.0 Hz, 1H), 2.81 – 2.73 (m, 1H), 2.58 (d, J = 12.2 Hz, 1H), 2.46 (d, J = 9.2 Hz, 4H), 2.40 – 2.34 (m, 5H), 2.17 (d, J = 7.7 Hz, 6H), 2.02 (dd, J = 20.0, 8.8 Hz, 3H), 1.86 – 1.73 (m, 4H), 1.73 – 1.56 (m, 11H), 1.50 (dt, J = 14.4, 7.3 Hz, 2H), 1.32 (s, 3H), 1.23 (s, 4H), 1.18 (s, 3H), 1.04 (d, J = 9.5 Hz, 6H), 0.91 – 0.80 (m, 1H), 0.32 (s, 3H).

^{13}C NMR (DMSO-d6, c = 1.1.10^{-2}M) δ 179.53, 178.25, 175.13, 173.97, 173.67, 173.25, 172.81, 172.53, 171.13, 170.98, 165.39, 164.66, 164.59, 156.16, 142.23, 136.25, 132.66, 131.91, 131.28, 129.77, 124.79, 120.36, 116.38, 111.42, 105.85, 103.08, 93.57, 85.94, 84.38, 79.48, 74.77, 72.92, 72.10, 70.40, 69.78, 69.55, 68.83, 68.09, 67.95, 63.14, 58.63, 54.95, 54.01, 52.97, 50.33, 48.76, 47.37, 46.55, 44.57, 42.09, 41.85, 40.20, 38.07, 37.59, 35.90, 35.13, 33.99, 31.66, 31.05, 29.89, 29.63, 29.27, 27.16, 25.79, 25.56, 20.09, 20.04, 19.93, 19.89, 19.84, 18.72, 16.51, 16.40, 15.65, 15.05.

4.3 Synthesis of B_{12}-nanogel 3

B_{12}-nanogels were synthesized via precipitation polymerization in a three-neck flask equipped with a reflux condenser under an inert atmosphere.

N-isopropylacrylamide (NIPA, 0.1901 g, 84 mM), acrylic acid (AA, 0.0137 mL, 10 mM), and *N,N'*-bis(acryloyl)cystamine (BAC, 0.0052 g, 1 mM, in 0.5 mL EtOH) were dissolved in deionized water (15 mL). The solution was heated to 55 °C and deoxygenated with Ar for 30 min under stirring (250 rpm).

Polymerization was initiated by addition of ammonium persulfate (APS, 0.009 g in 1 mL H_2O) and TEMED (7 µL). After a few minutes, the B_{12}-monomer **2** (0.1656 g, 5 mM, in 4 mL H_2O) was added. The reaction was allowed to proceed for 4 h and then quenched by cooling to room temperature.

The crude product was purified by dialysis (MWCO 10 kDa) against deionized water (pH 3.0 for 2 h, followed by pH 7.0 for 24 h), repeated three times to remove unreacted species. The purified nanogel suspension was centrifuged (20 000 × g, 10 min, 23 °C). The absence of residual B_{12} species in the supernatant was confirmed visually and by

UV–Vis spectroscopy yielding approximately 15 mg mL^{-1} for B_{12}-nanogels **3**. The B_{12} content was estimated by UV–Vis spectroscopy based on the difference between the initial amount and the remaining unbound fraction after purification, yielding 0.1524 g (7.62 mg mL^{-1}, 92%).

4.4 Synthesis of control gel

Control nanogels were prepared under identical conditions, omitting monomer **2** yielding final concentrations (based on dry mass) of 5 mg mL^{-1}.

5. Analytical Data

5.1 Chromatograms and Optical spectra

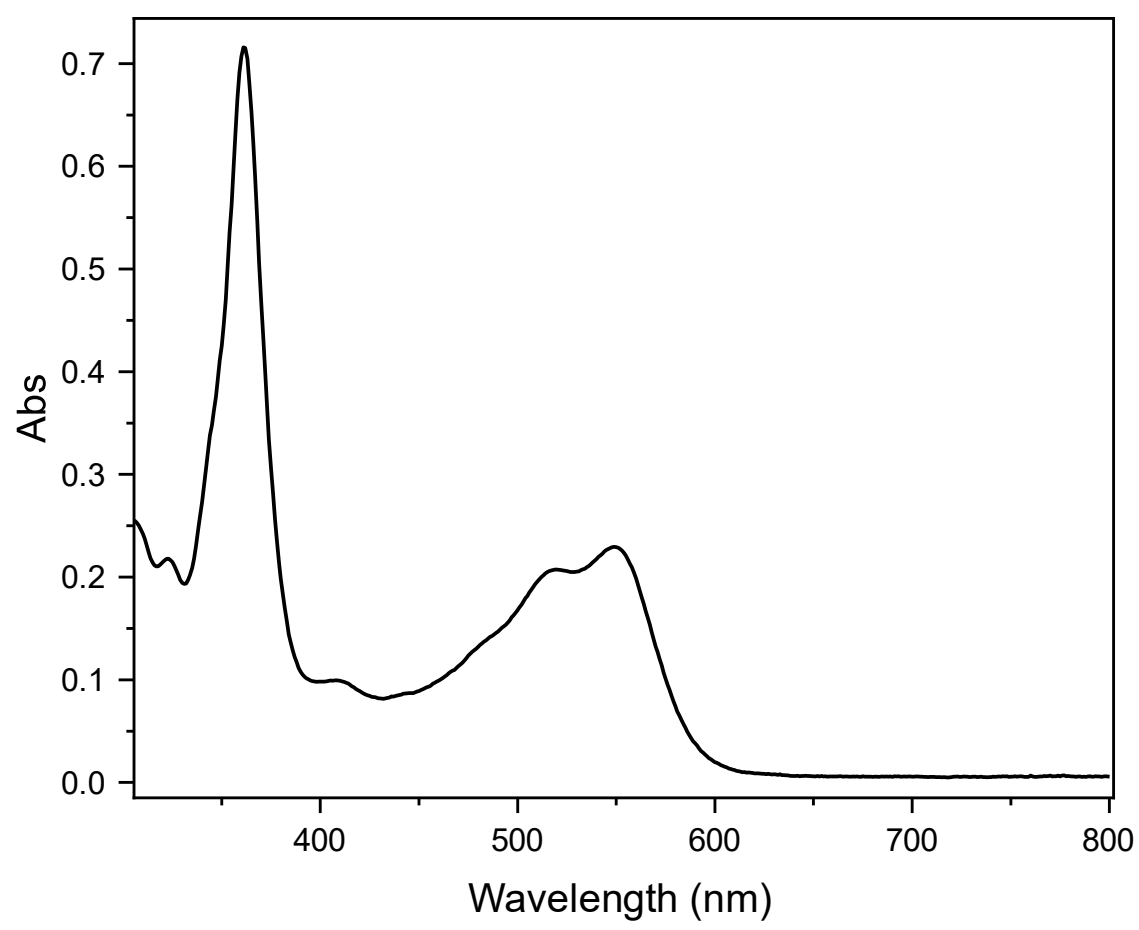

Figure S1. UV-Vis spectrum of **5’-B$_{12}$-PEG$_3^+$ trifluoroacetate** (H_2O, c = 4.0·10^{-5} M).

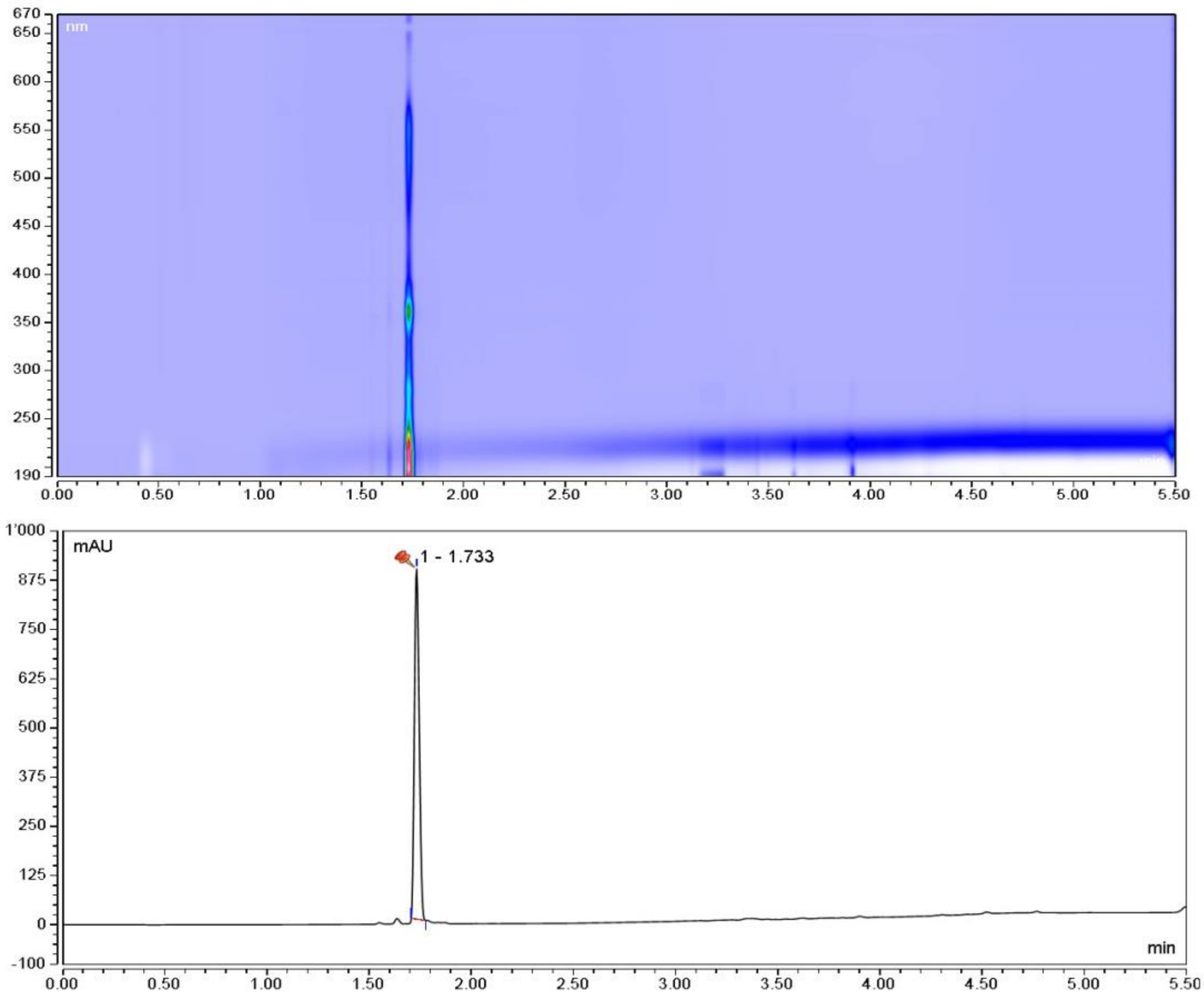

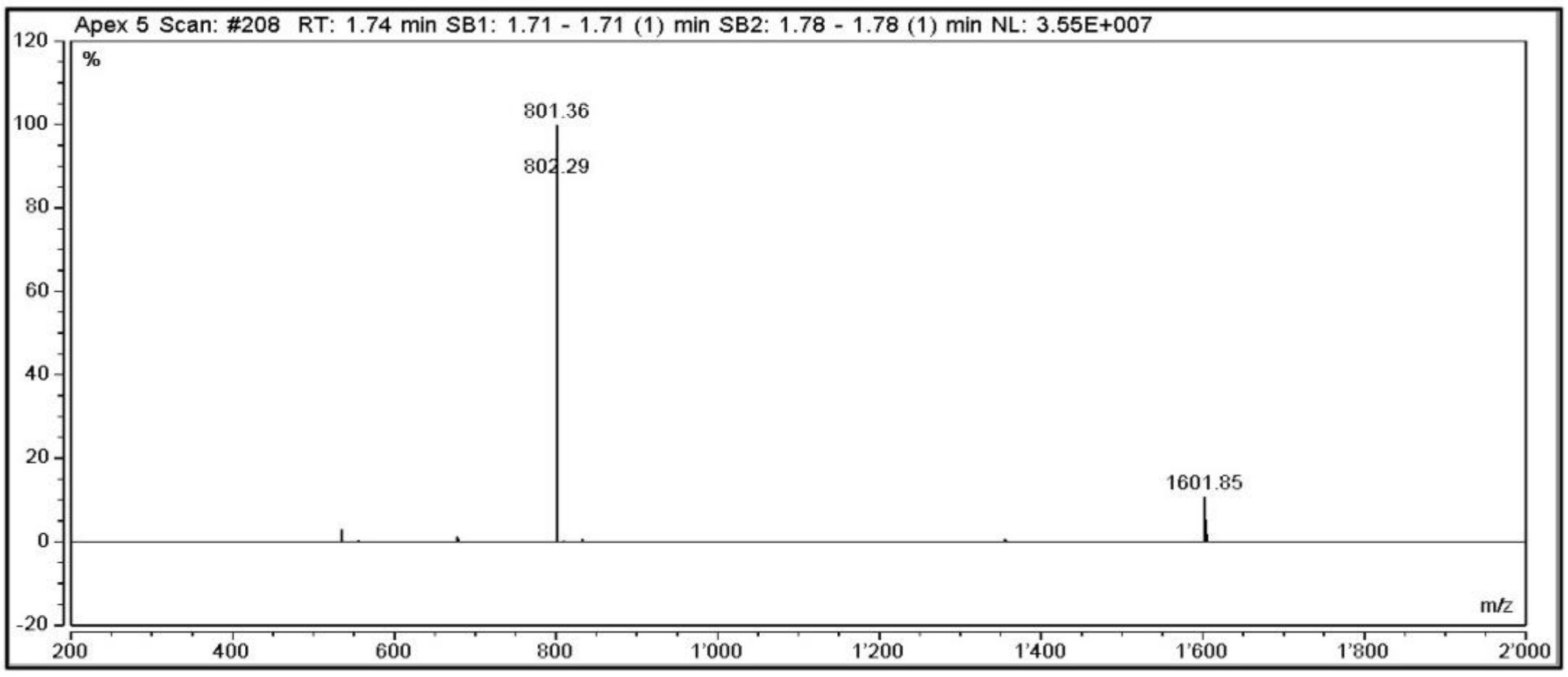

Figure S2. UPLC-MS of **5’-B_{12}-PEG_3^+ trifluoroacetate**, Top: UV-Vis vs time chromatogram, center: UV chromatogram (270 nm) (t_{ret} = 1.733 min) and bottom: ESI-MS spectrum.

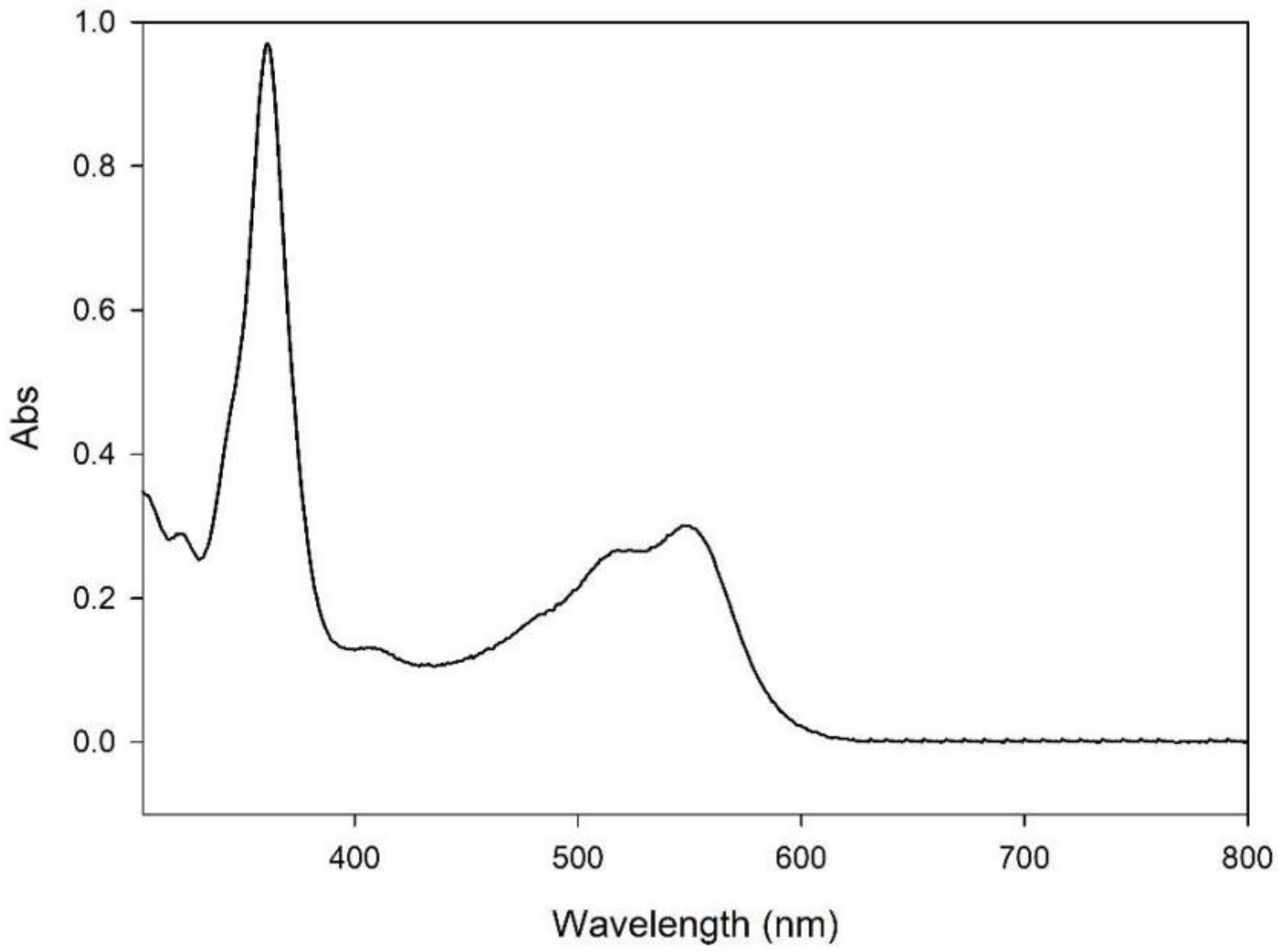

Figure S3. UV-Vis spectrum of **B_{12}-monomer** (**2**, H_2O, c = $4.0 \cdot 10^{-5}$ M).

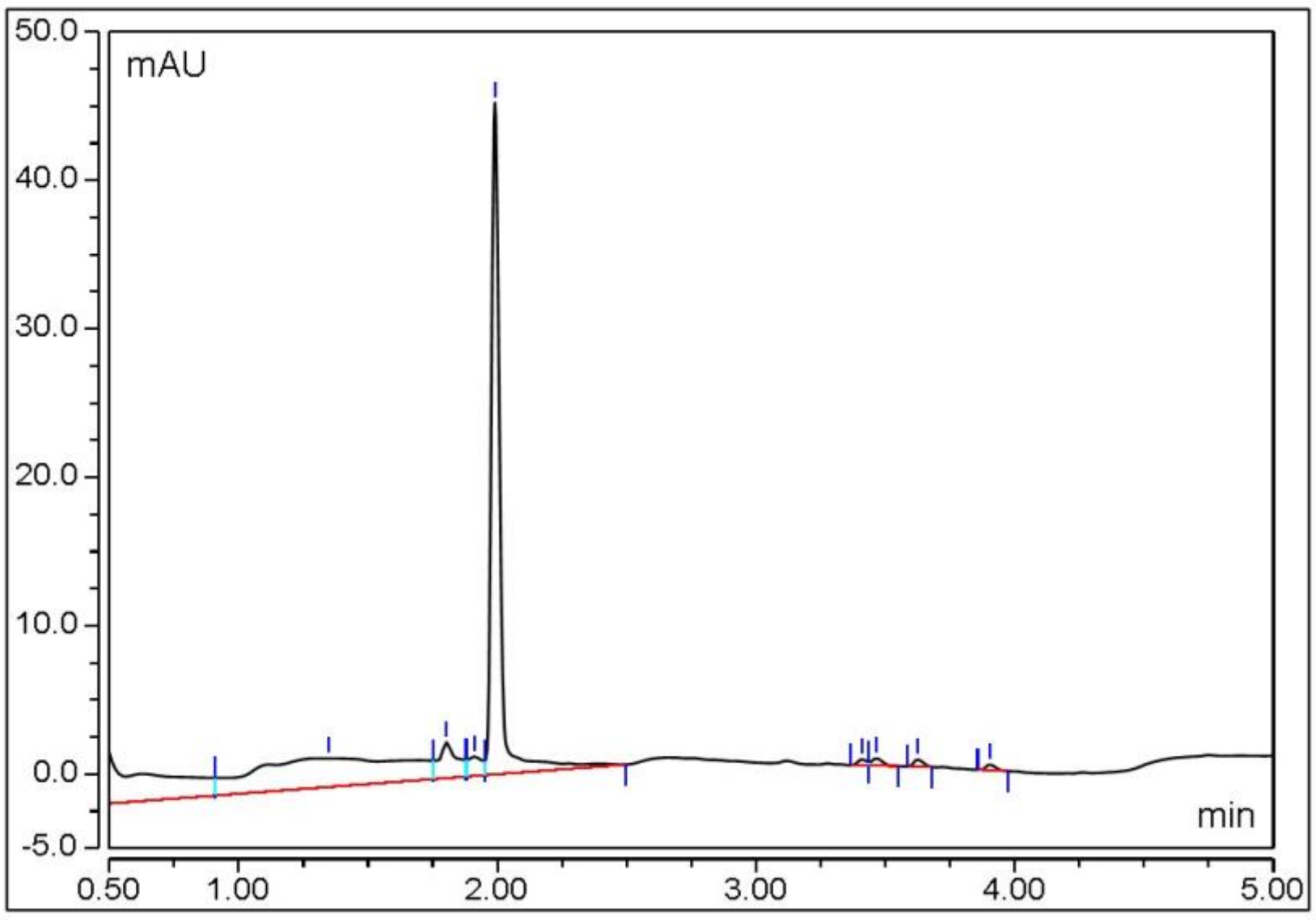

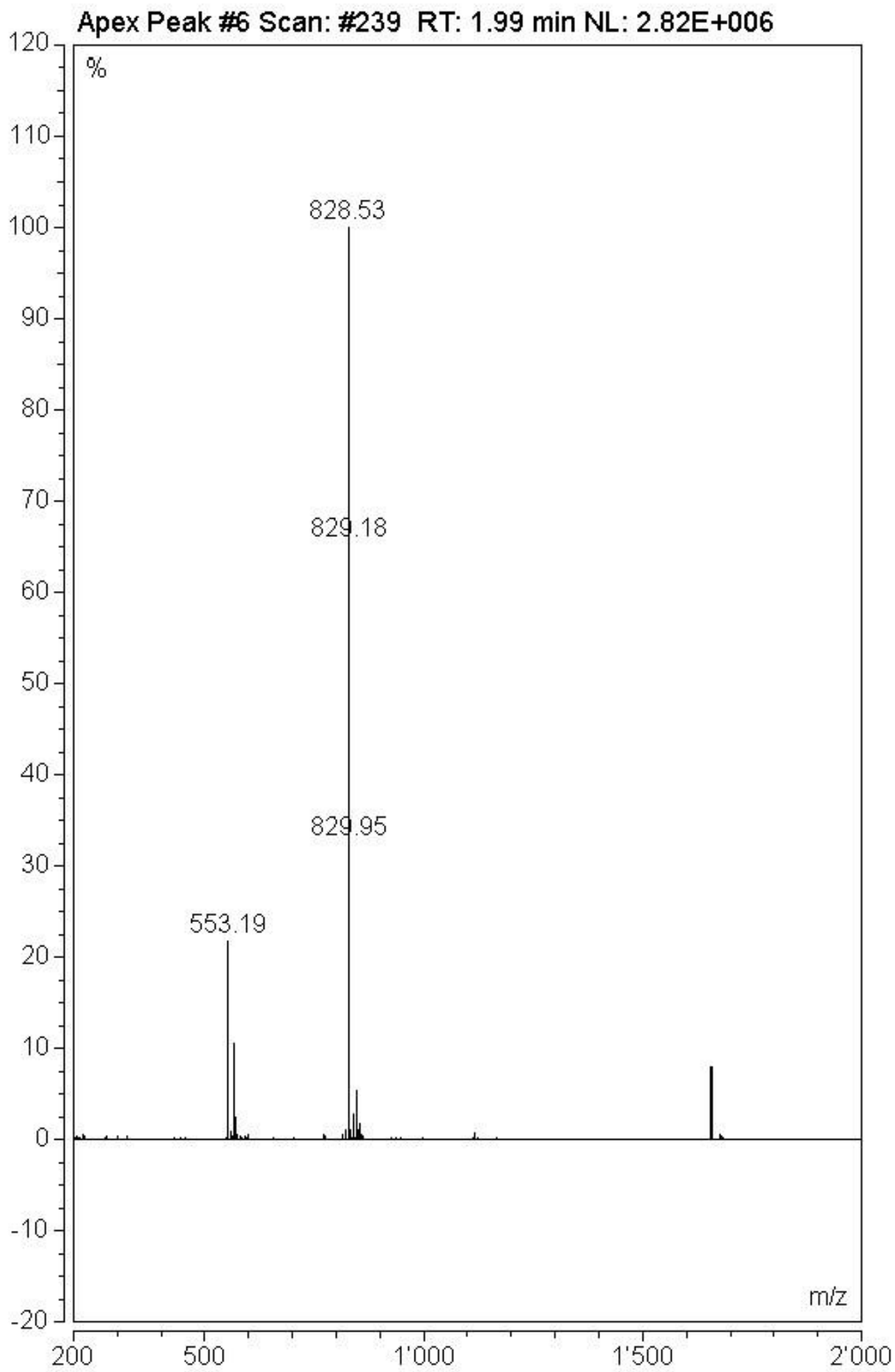

Figure S4. UPLC-MS of **B_{12}-monomer 2**, Top: UV chromatogram (280 nm) (t_{ret} = 1.99 min) and bottom: ESI-MS spectrum.

5.2 NMR spectra, table of NMR data and discussion.

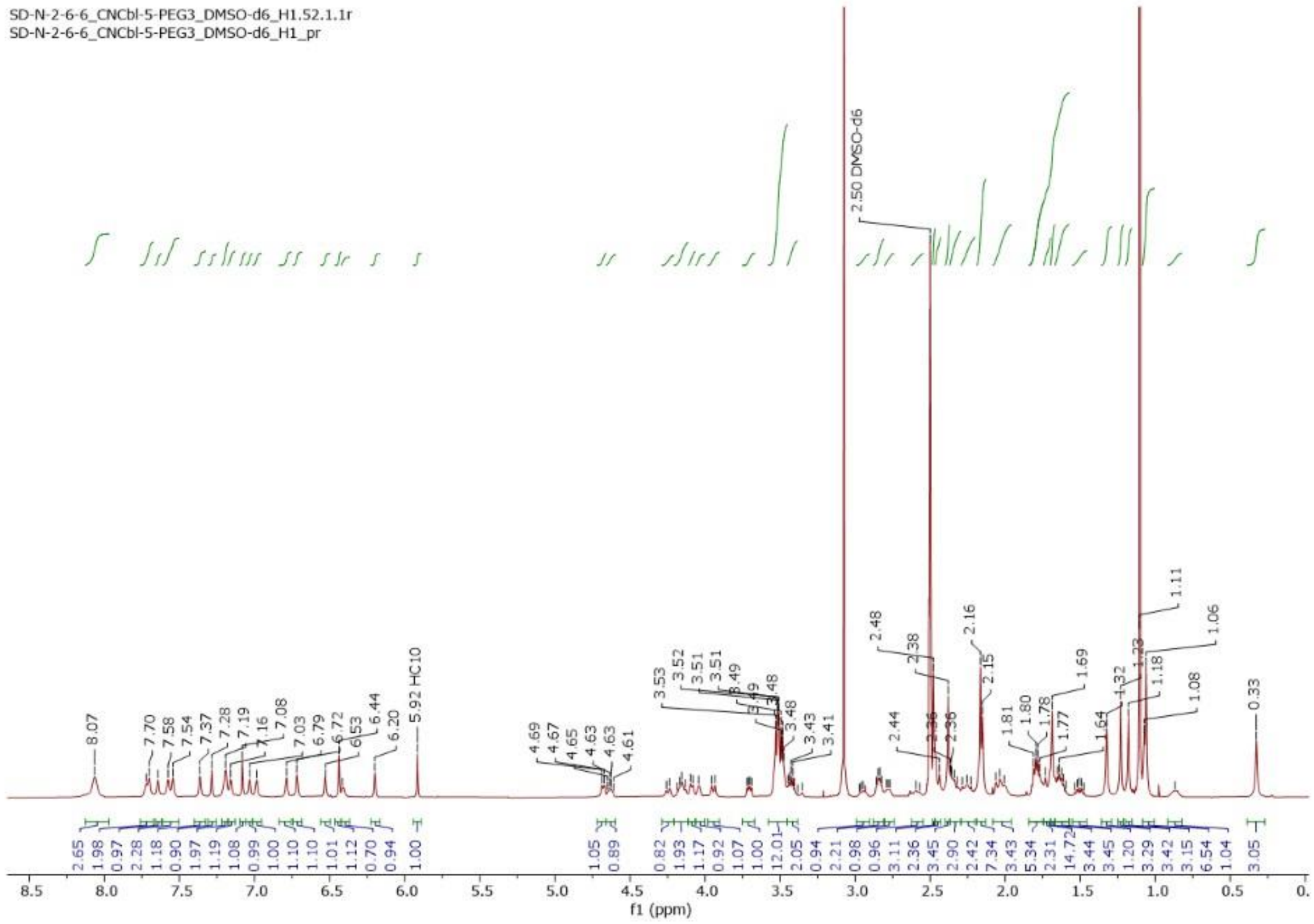

Figure S5. ^{1}H NMR spectrum of **5'-B12-PEG3+ trifluoroacetate** (500 MHz, DMSOd6, 298 K, $1.2\cdot10^{-2}$ M).

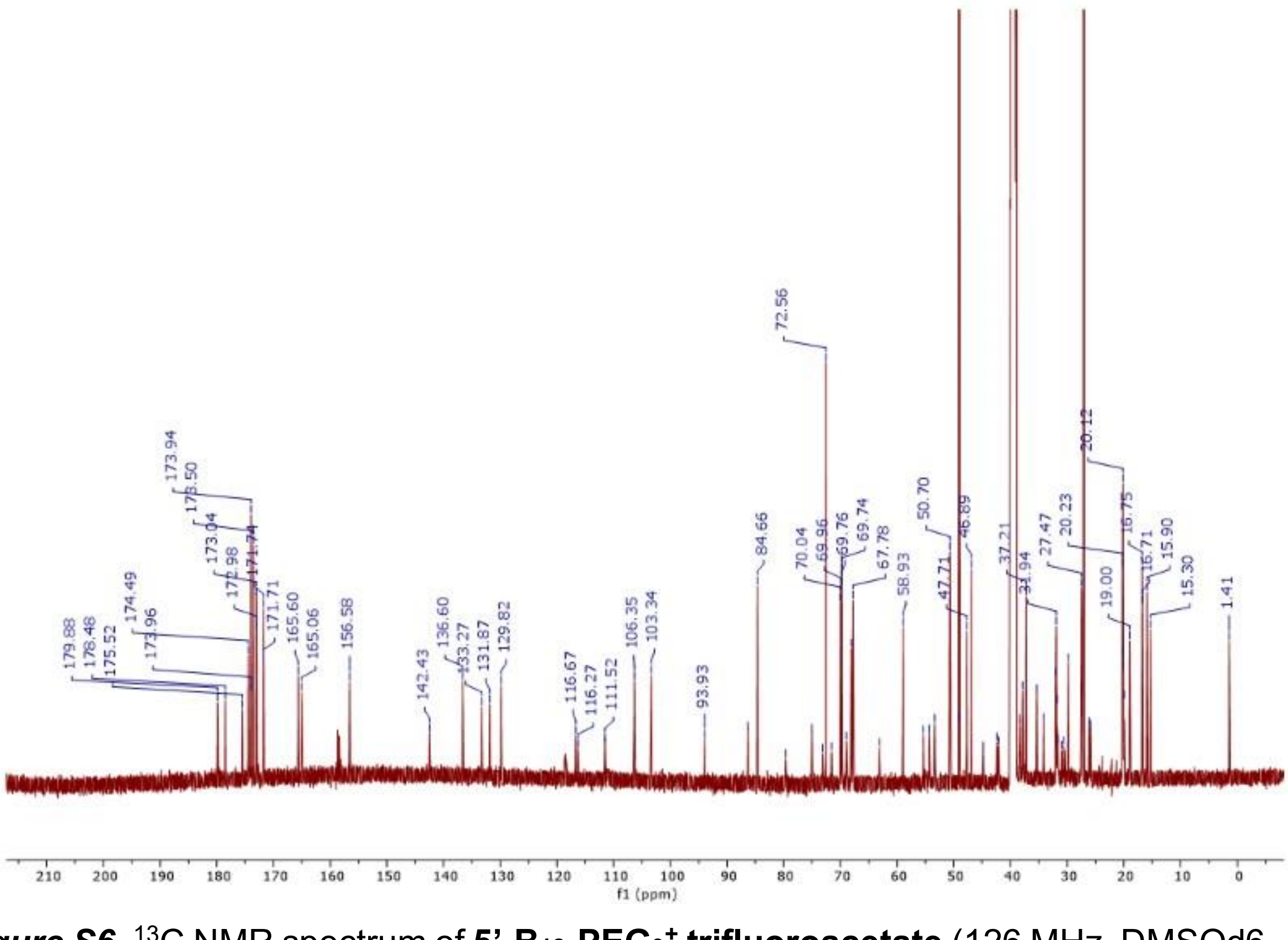

Figure S6. ^{13}C NMR spectrum of **5'-B12-PEG3+ trifluoroacetate** (126 MHz, DMSOd6, 298 K, $1.2\cdot10^{-2}$ M).

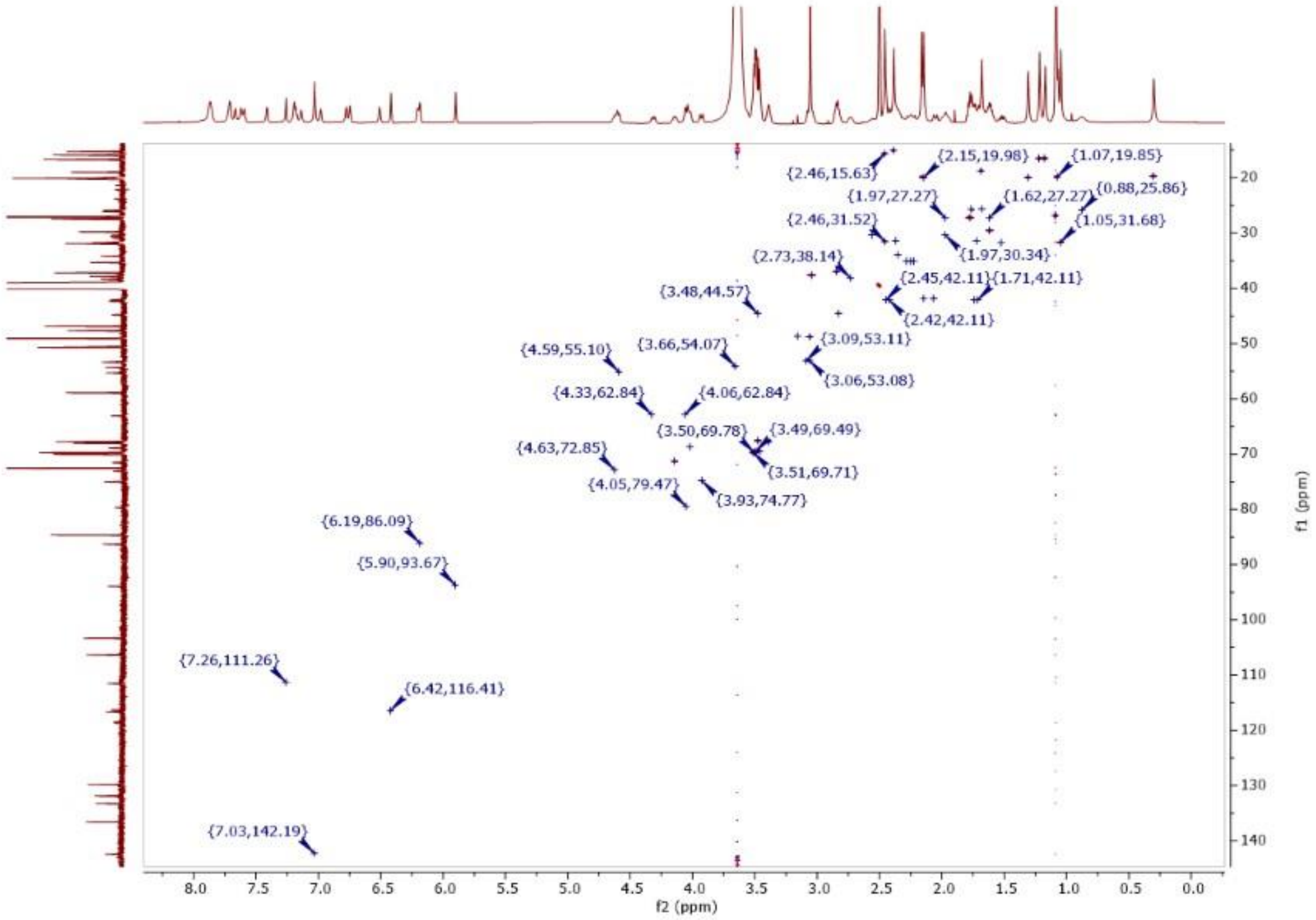

***Figure S7*.** ^{1}H ^{13}C HSQC spectrum of **5'-B$_{12}$-PEG$_3^+$ trifluoroacetate** (500 MHz, 126 MHz, DMSO-d6, 298 K, 1024 x 4096 p, 4 scans, 1.2·10^{-2} M).

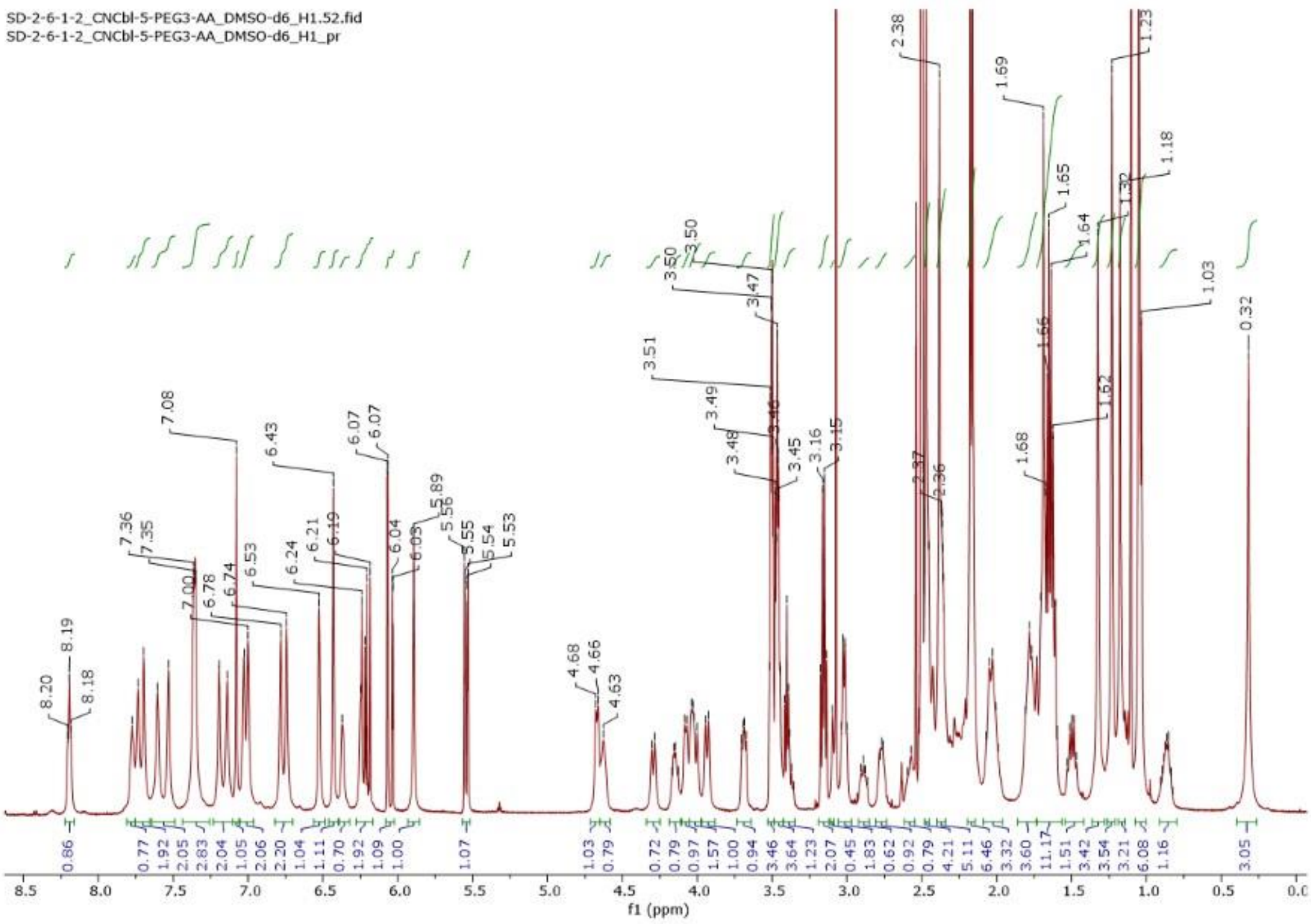

***Figure S8*.** ^{1}H NMR spectrum of B$_{12}$-monomer **2** (500 MHz, DMSO-d6, 298 K,

1.1·10^{-2} M).

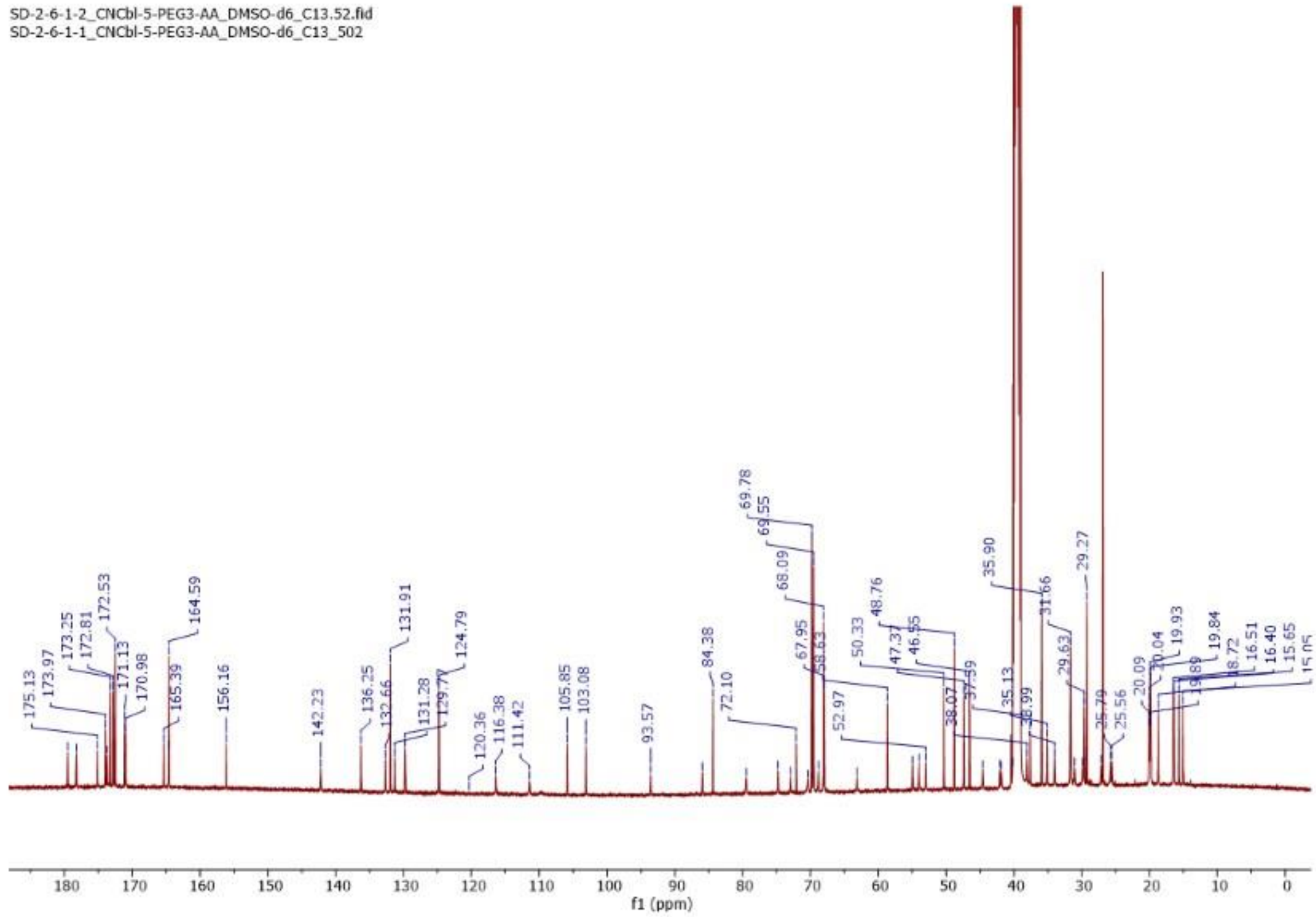

Figure S9. ^{13}C NMR spectrum of B_{12} monomer **2** (126 MHz, DMSO-d6, 298 K, 1.1·10^{-2} M).

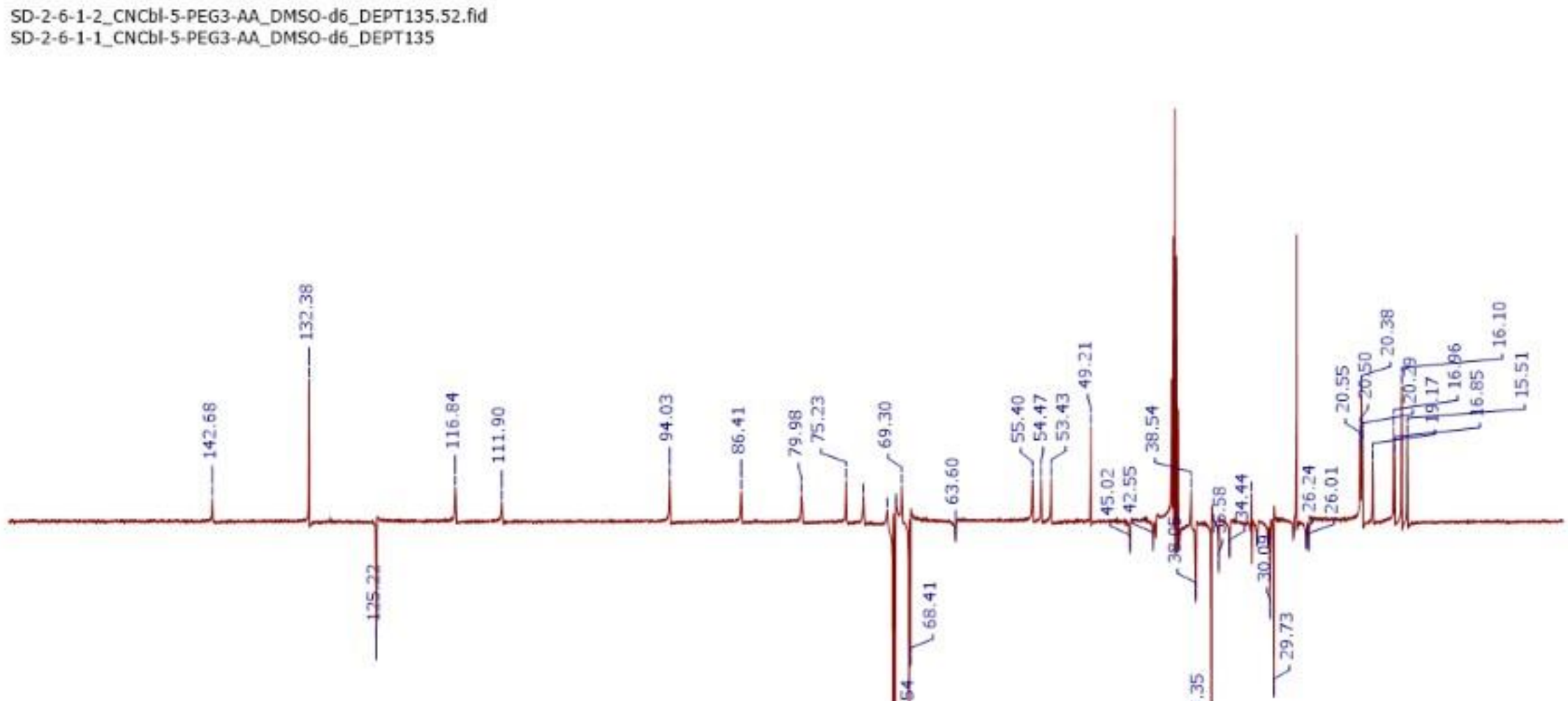

Figure S10. DEPT 135-NMR spectrum of B_{12} monomer **2** (500 MHz, DMSO-d6, 298 K, 1.1·10^{-2} M).

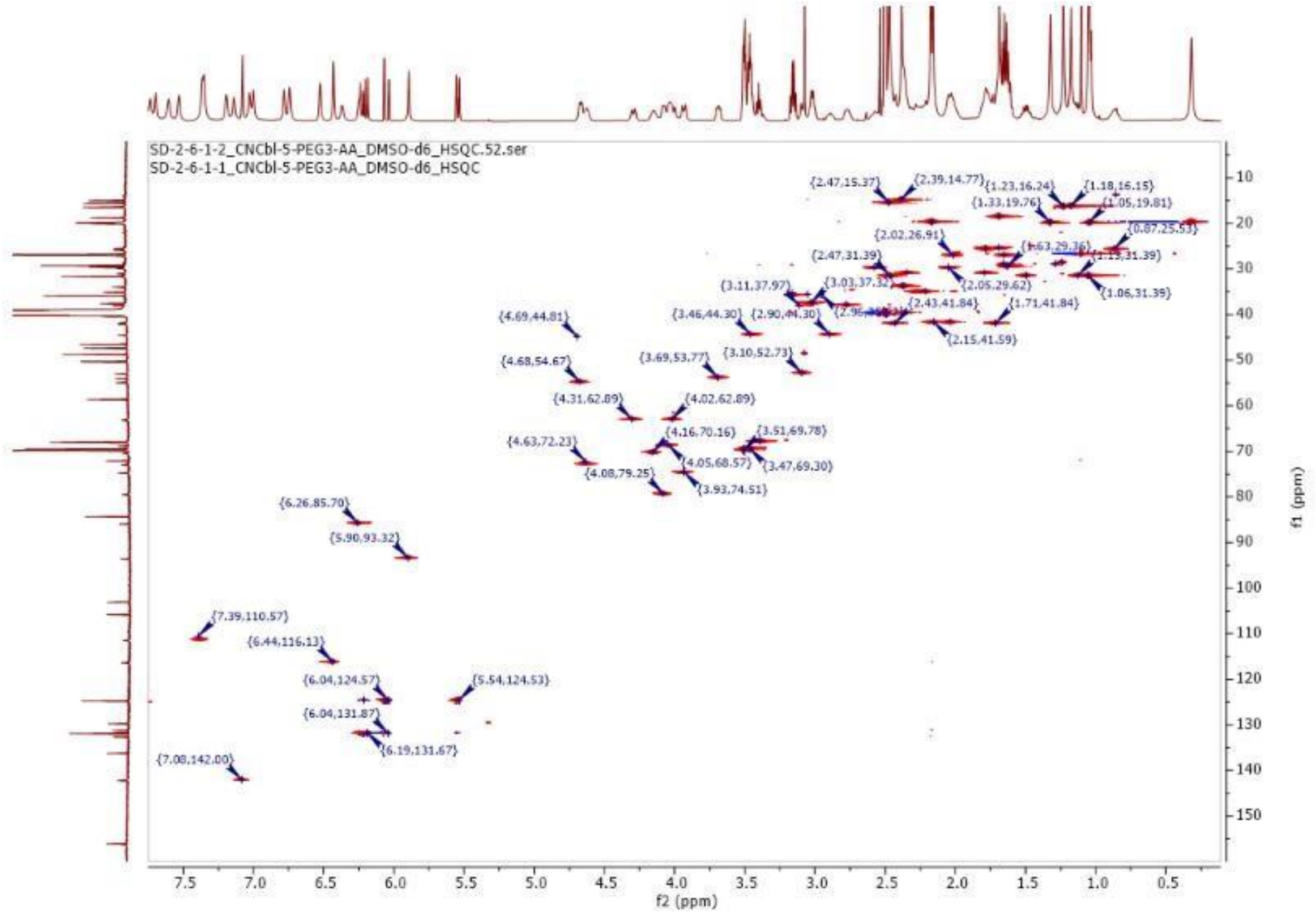

Figure S11. 1H ^{13}C HSQC spectrum of B_{12}-monomer **2** (500 MHz, 126 MHz, DMSO-d6, 298 K, 1024 x 4096 p, 10 scans, 1.1·10^{-2} M).

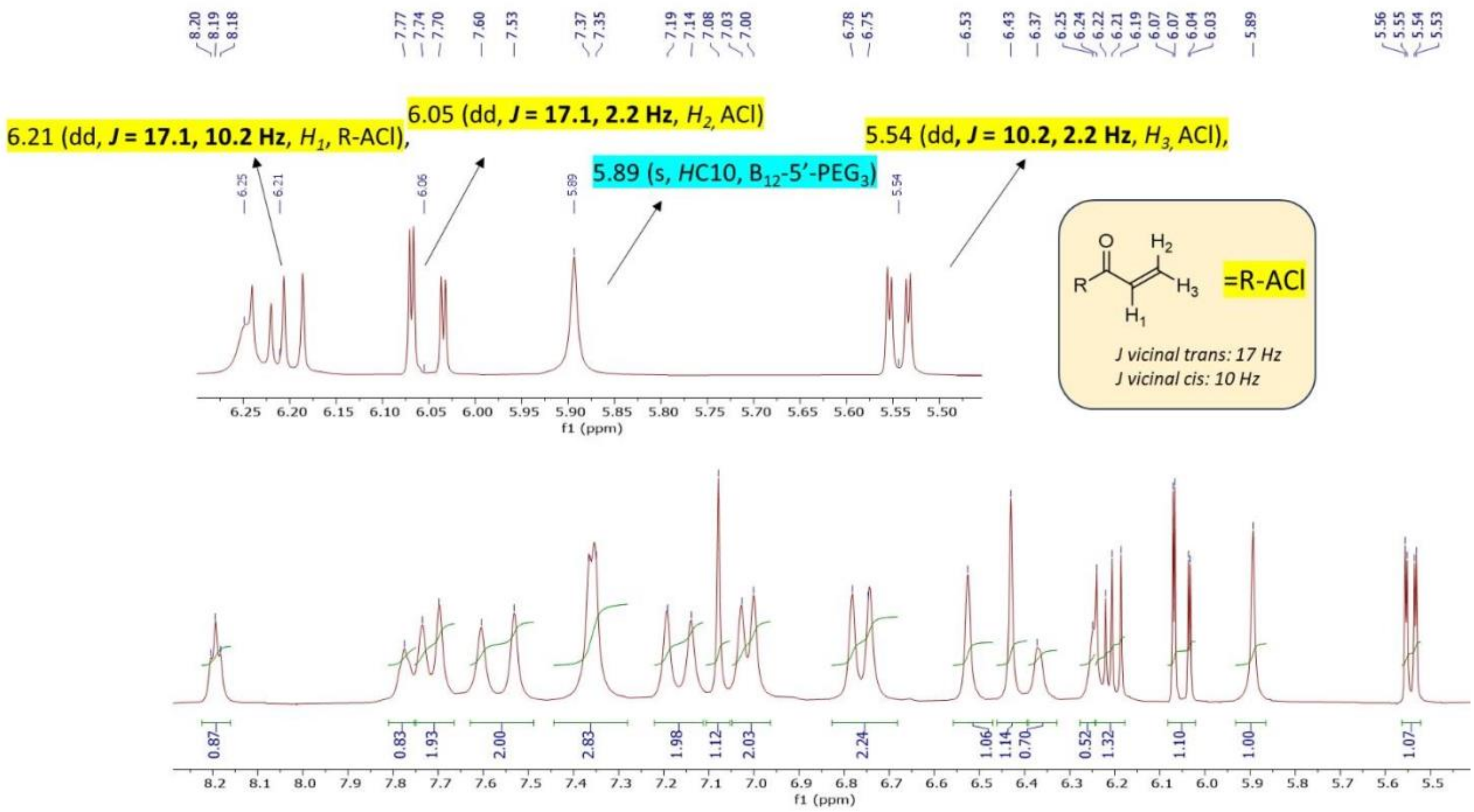

Figure S12. Section of the ^{1}H NMR of the spectra for B_{12}-monomer **2** depicting the key NMR signals of the acryloyl chloride unit (R-ACl) along with the characteristic signals of the CNCbl-5'-PEG_3-ACl unit.

5.3 Nanogel Size and Morphology

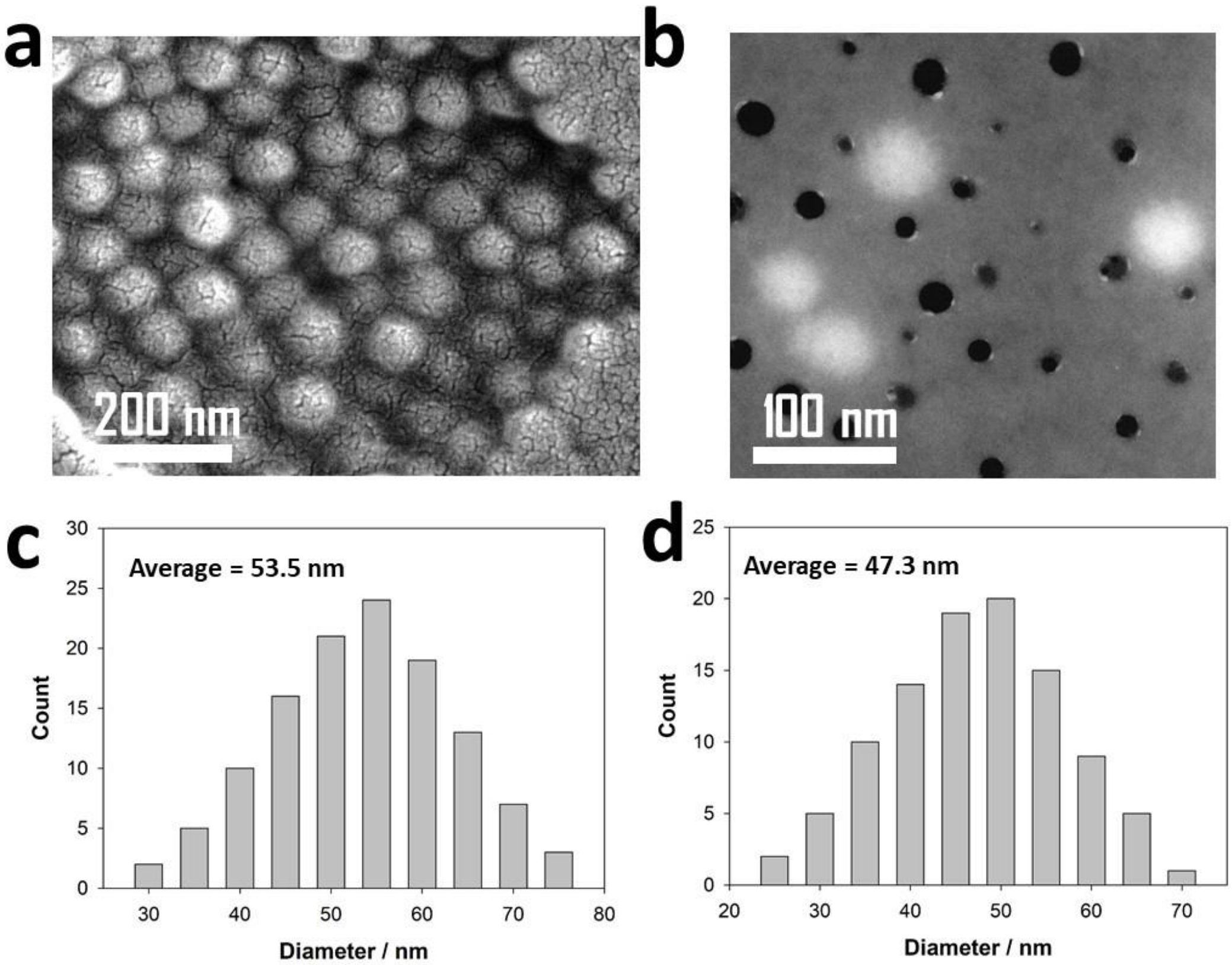

Figure S13. (a) SEM and (b) TEM images of the B_{12}-nanogel **3**. Corresponding particle size distribution histograms determined from (c) SEM and (d) TEM image analysis

Table S1. Size distributions at 37 °C and surface potentials pH 7.4 of nanogels.

	Diameter (nm)	Polydispersity Index (PDI)	Zeta Potential (mV)
**Control-nanogel**	102 ± 1.0	0.11 ± 0.01	-17.0 ± 0.8
**B_{12}-nanogel 3**	119 ± 1.4	0.23 ± 0.01	-18.5 ± 0.6

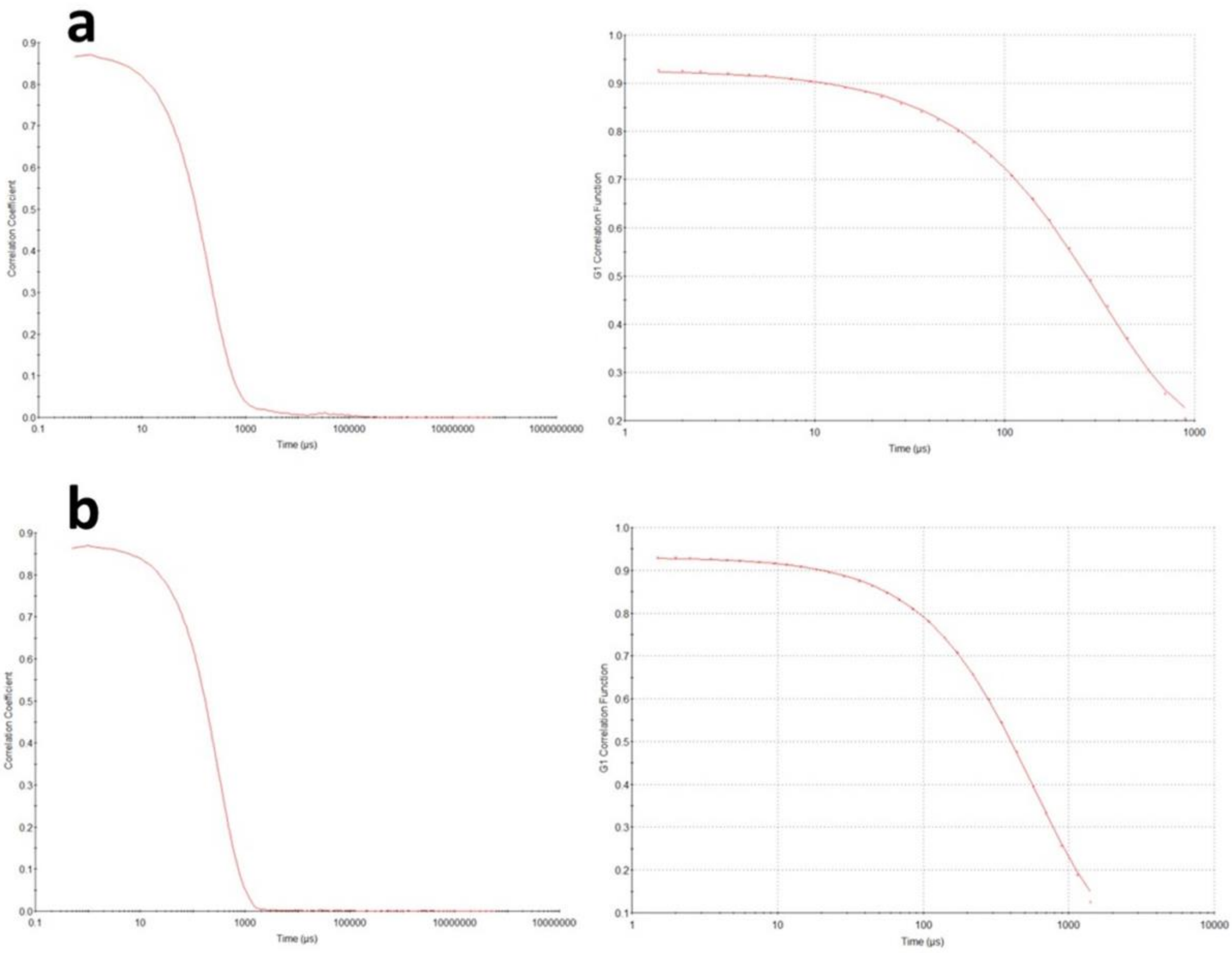

Figure S14. Representative DLS correlation functions for (a) control nanogel and (b) B_{12}-nanogel **3**, showing the experimental correlation data (left) and corresponding cumulants fits (*right*).

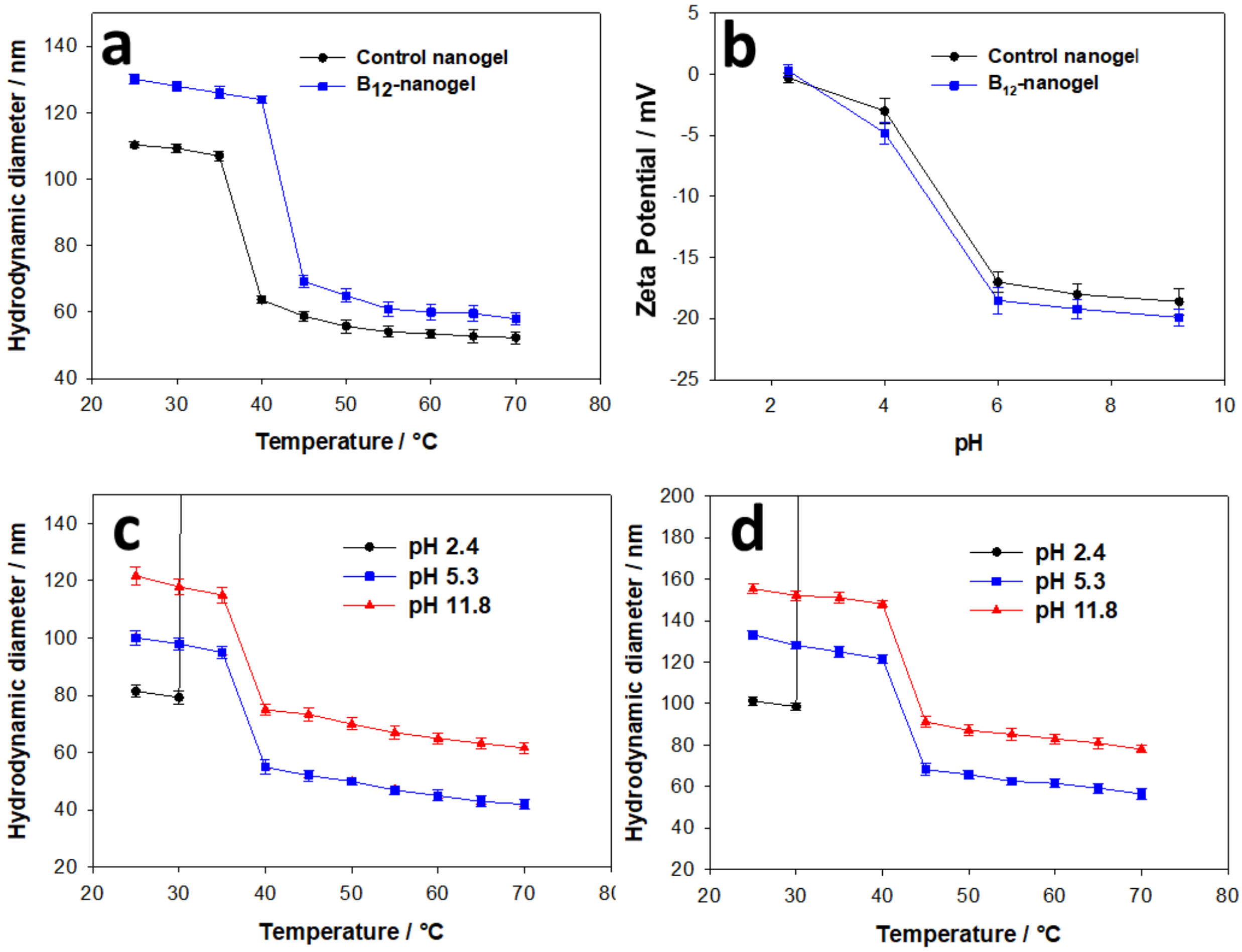

Figure S15. (a) Hydrodynamic diameter as a function of temperature (pH 6). (b) Zeta potential as a function of pH for the control-, and B_{12}-nanogels. (c) Temperature dependent hydrodynamic diameter variations at different pH levels for the control nanogel. (d) Temperature-dependent hydrodynamic diameter variations at different pH levels for the B_{12}-nanogel. Error bars represent the standard deviation of three independent measurements (mean ± SD, n = 3).

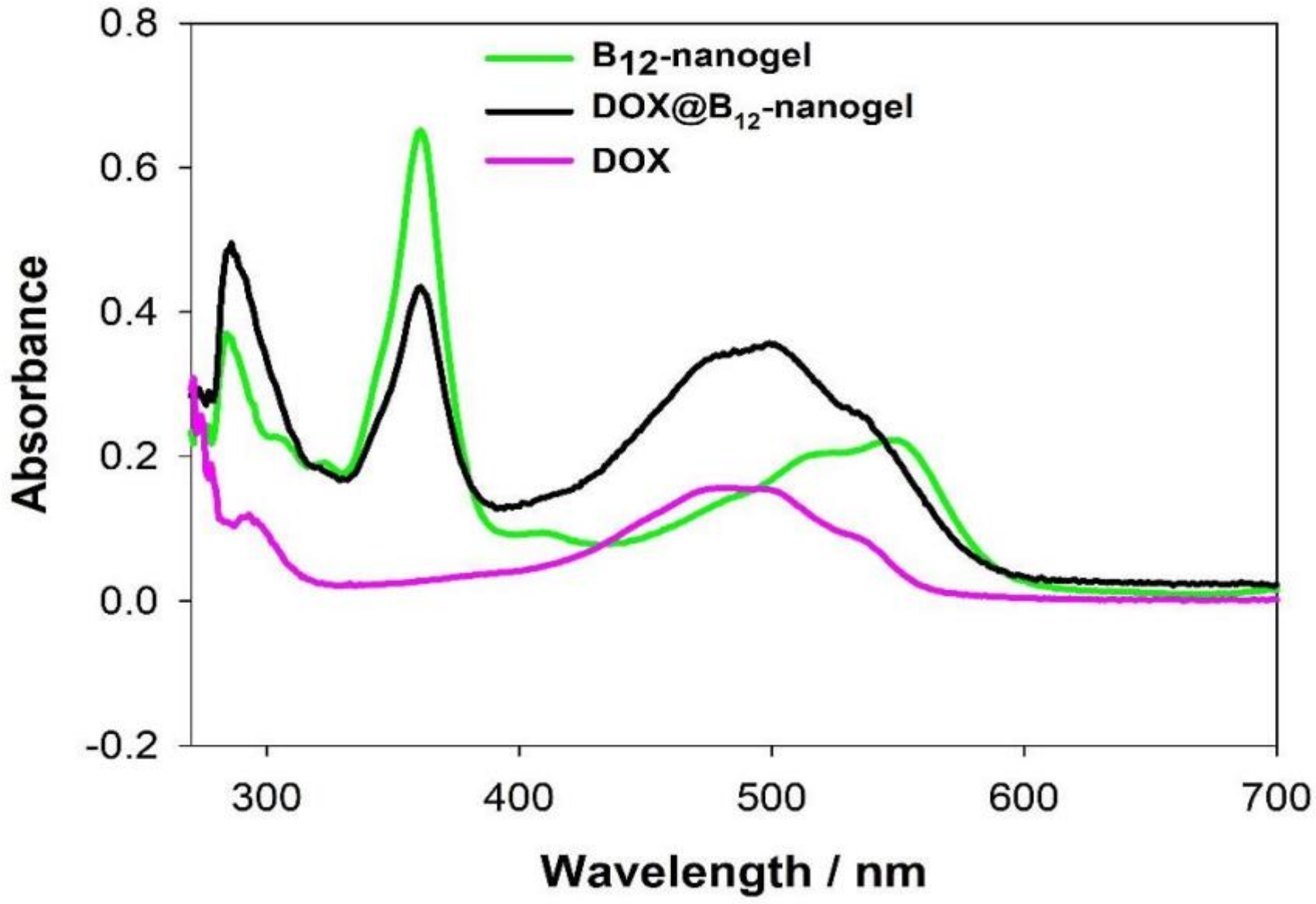

Figure S16. UV-Vis absorption spectra of B_{12}-nanogels, DOX@B_{12}-nanogels and DOX.

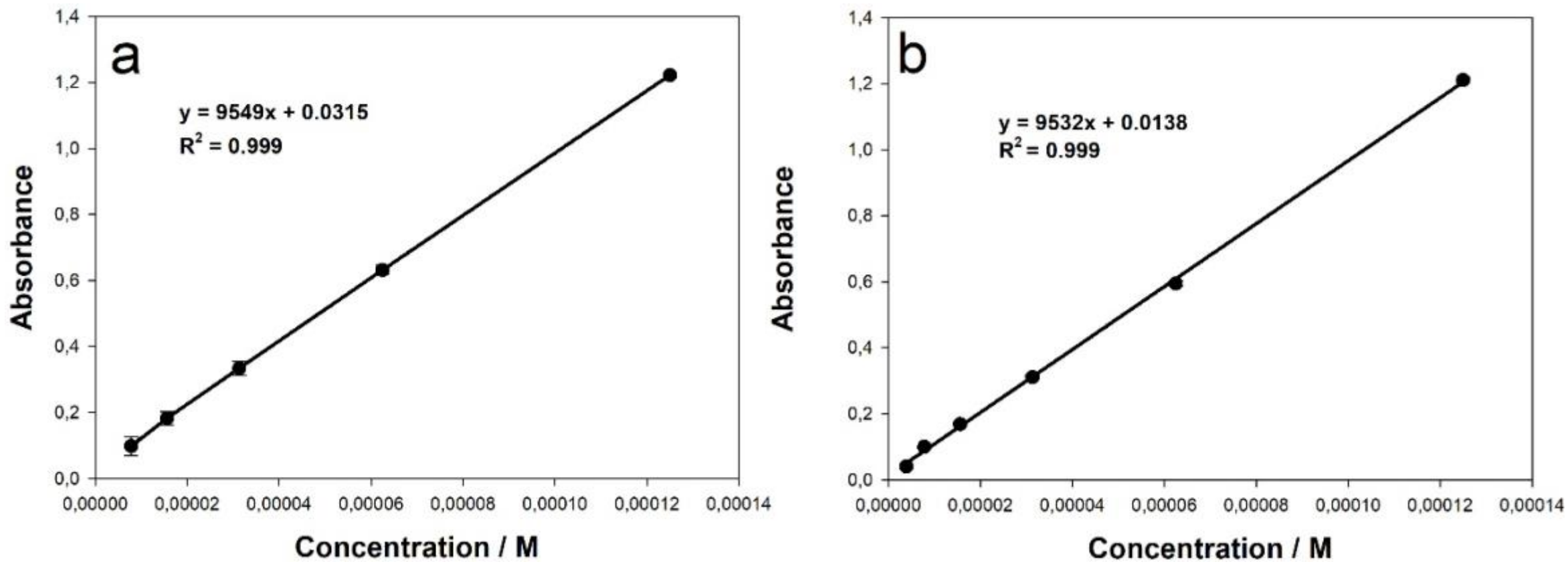

Figure S17. Calibration curves as function of absorbance at 480 nm against concentration of DOX in (a) acetate buffer (pH 5.0, 0.1 M) and (b) HEPES buffer (pH 7.4, 0.1 M). Linear regression equations and R^2 values are shown in the corresponding plots. Data are presented as mean ± SD (n = 3).

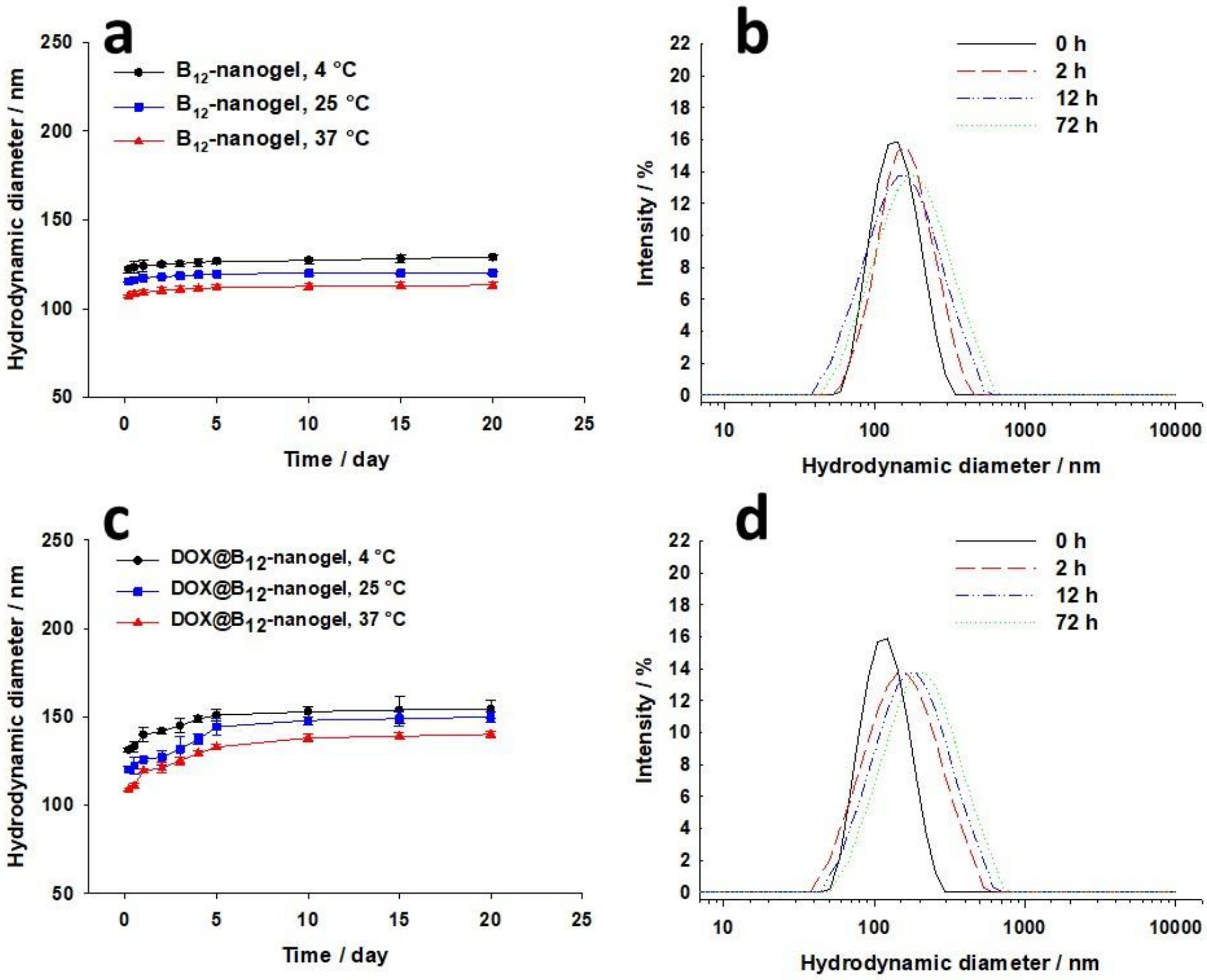

Figure S18. *Hydrodynamic diameter changes plotted vs. time at pH 7.4 (HEPES buffer, 0.1 M) at 4 °C, 25 °C and 37 °C and mean diameter distribution changes with time, in the presence of fetal bovine serum (FBS, 10 mM) at 37 °C for* B_{12}*-nanogel* **3** *(a, b) and DOX@*B_{12}*-nanogel* **4** *(c, d).*

5.4 Cytotoxicity data

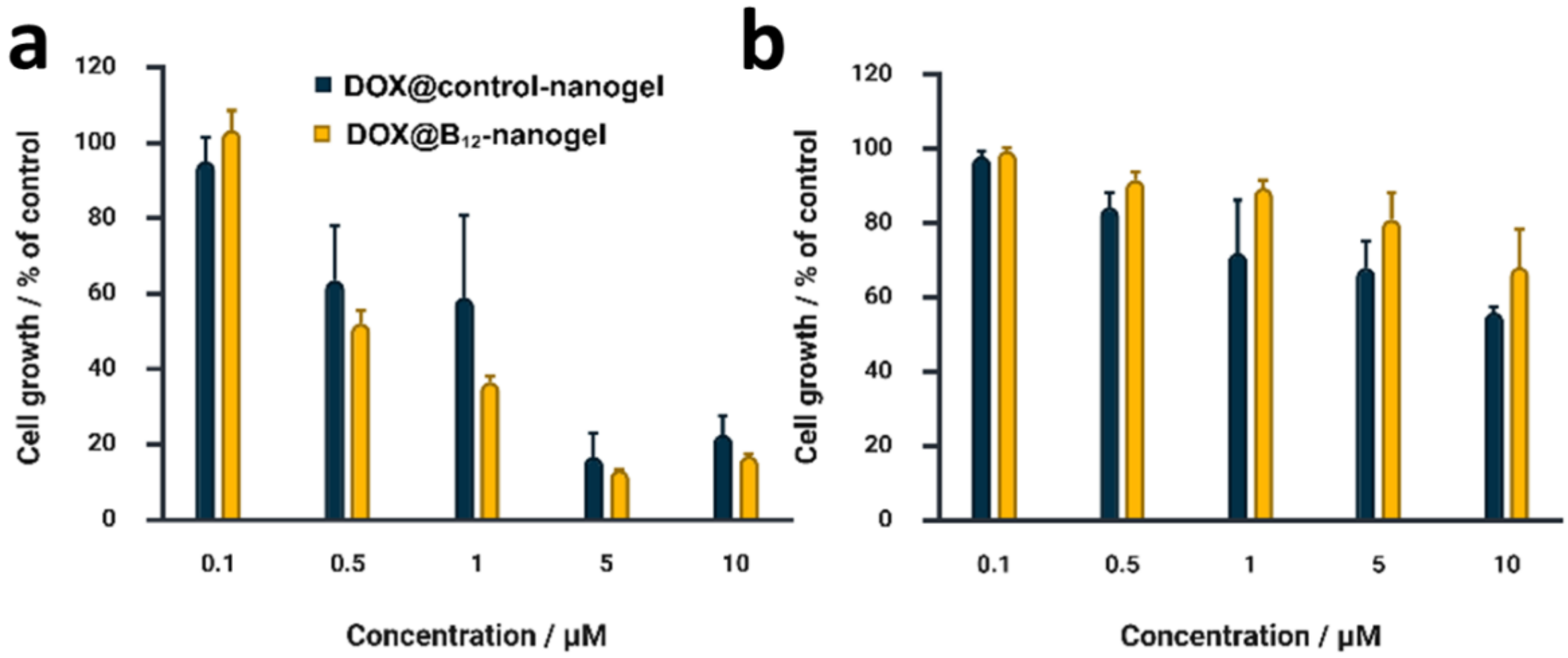

Figure S19. Results of MTT assay using (a) HT-29 and (b) CCD 841 CoN cell lines after 72 h treatment with DOX@control-nanogel and DOX@B_{12}-nanogel at concentrations between 0.1 and 10 μM.

References

[1] R. A. Horton, J. D. Bagnato, and C. B. Grissom, J. Org. Chem. 2003, 68, 7108–7111.
